# Luminescence and Light‐Driven Energy and Electron Transfer from an Exceptionally Long‐Lived Excited State of a Non‐Innocent Chromium(III) Complex

**DOI:** 10.1002/anie.201909325

**Published:** 2019-10-31

**Authors:** Steffen Treiling, Cui Wang, Christoph Förster, Florian Reichenauer, Jens Kalmbach, Pit Boden, Joe P. Harris, Luca M. Carrella, Eva Rentschler, Ute Resch‐Genger, Christian Reber, Michael Seitz, Markus Gerhards, Katja Heinze

**Affiliations:** ^1^ Institute of Inorganic Chemistry and Analytical Chemistry Johannes Gutenberg University of Mainz Duesbergweg 10–14 55128 Mainz Germany; ^2^ Division Biophotonics Federal Institute for Materials Research and Testing (BAM) Richard-Willstätter-Straße 11 12489 Berlin Germany; ^3^ Institute of Chemistry and Biochemistry Freie Universität Berlin Takustraße 3 14195 Berlin Germany; ^4^ Institute of Inorganic Chemistry University of Tübingen Auf der Morgenstelle 18 72076 Tübingen Germany; ^5^ Department of Chemistry and Research Center Optimas TU Kaiserslautern Erwin-Schrödinger-Straße 67663 Kaiserslautern Germany; ^6^ Département de chimie Université de Montréal Montréal Québec H3C 3J7 Canada

**Keywords:** Earth-abundant metals, Laporte's rule, Luminescence, Photoredox chemistry, Sustainable Chemistry

## Abstract

Photoactive metal complexes employing Earth‐abundant metal ions are a key to sustainable photophysical and photochemical applications. We exploit the effects of an inversion center and ligand non‐innocence to tune the luminescence and photochemistry of the excited state of the [CrN_6_] chromophore [Cr(tpe)_2_]^3+^ with close to octahedral symmetry (tpe=1,1,1‐tris(pyrid‐2‐yl)ethane). [Cr(tpe)_2_]^3+^ exhibits the longest luminescence lifetime (*τ*=4500 μs) reported up to date for a molecular polypyridyl chromium(III) complex together with a very high luminescence quantum yield of *Φ*=8.2 % at room temperature in fluid solution. Furthermore, the tpe ligands in [Cr(tpe)_2_]^3+^ are redox non‐innocent, leading to reversible reductive chemistry. The excited state redox potential and lifetime of [Cr(tpe)_2_]^3+^ surpass those of the classical photosensitizer [Ru(bpy)_3_]^2+^ (bpy=2,2′‐bipyridine) enabling energy transfer (to oxygen) and photoredox processes (with azulene and tri(*n*‐butyl)amine).

## Graphical abstract

**Super‐long luminescence lifetime** (4500 μs) at room temperature, high quantum yield (8.2 %) and reversible redox chemistry in the complex [Cr(tpe)_2_]^3+^ containing an Earth‐abundant first row transition metal ion is enabled by ligand bite angle, inversion symmetry and ligand non‐innocence (tpe=1,1,1‐tris (pyrid‐2‐yl)ethane). [Cr(tpe)_2_]^3+^ is competent in light‐induced energy and electron transfer processes similar to classical noble metal ion complexes.

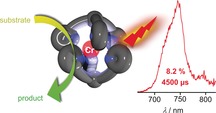

## Introduction

**Scheme 1:**
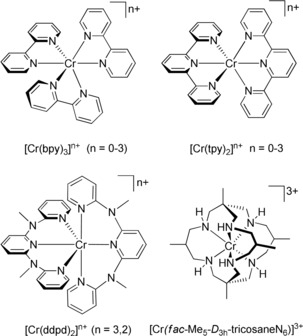
Selected luminescent chromium(III) complexes (*n*=3) and their reduced counterparts (*n*=2, 1, 0).

A strongly growing interest in chromium(III) complexes, especially with polypyridyl ligands, arises from two perspectives, namely from the ambiguity of the ground state electronic structures of their reduced congeners (redox non‐innocence)^1^, ^2^ and their—for first row transition metal complexes—outstanding luminescent properties with long‐lived spin‐flip emission from doublet states.^3^, ^4^, ^5^ The type of polypyridine ligand determines both, redox and photophysical properties of chromium(III) complexes. The classical electron transfer series [Cr(^t^bpy)_3_]^*n*+^ and [Cr(tpy)_2_]^*n*+^ (*n*=3, 2, 1, 0) exclusively comprise ligand‐centered redox couples and the chromium center retains its oxidation state +III throughout (^t^bpy=4,4′‐di‐*tert*‐butyl‐2,2′‐bipyridine, tpy=2,2′:6′,2′′‐terpyridine; Scheme 1).^1^ Analogous results have been obtained for [Cr(^Me^PDP)_2_]^*n*−^ (H_2_^Me^PDI=2,6‐bis(5‐methyl‐3‐phenyl‐1*H*‐pyrrol‐2‐yl)pyridine) complexes.^2^ On the other hand, the [Cr(ddpd)_2_]^3+/2+^ redox couple featuring the electron‐rich polypyridine ligand ddpd involves a purely metal centered process giving chromium(II) (ddpd=*N*,*N*′‐dimethyl‐*N*,*N*′‐dipyridin‐2‐ylpyridine‐2,6‐diamine; Scheme 1).^3^, ^6^

Bis(terpyridine)chromium(III) [Cr(tpy)_2_]^3+^ and other classical pyridine complexes are weakly emissive (Table 1).^7^, ^8^, ^9^, ^10^, ^11^, ^12^, ^13^ Although electron donating substituents at the tpy ligands enhance absorption in the visible spectral region by intraligand charge transfer absorptions, luminescence quantum yields and lifetimes remain poor (Table 1).^14^, ^15^ Prior to our work on the strongly emissive complex [Cr(ddpd)_2_]^3+^ (Scheme 1, Table 1)^3^ featuring six‐membered chelate rings and hence N‐Cr‐N angles close to 90°, the highest luminescence lifetimes were reported for the hexaamine quasi‐cage and cage complexes [Cr(TAP[9]aneN_3_)]^3+^ and [Cr(*fac*‐Me_5_‐*D*_3*h*_‐tricosaneN_6_)]^3+^ (Table 1).^16^, ^17^, ^18^ Note, that these two ligands form six‐membered chelate rings with the chromium ion as well. Chromium(III) cage complexes with five‐membered chelate rings show shorter luminescence lifetimes and lower quantum yields.^19^ Deuteration of the ddpd ligand boosts the key luminescence data of [Cr(ddpd)_2_]^3+^ to *Φ*=30.0 % and *τ*=2300 μs in deaerated CD_3_CN (Table 1).^20^ These favorable photophysical data of [Cr(ddpd)_2_]^3+^ enable its application in temperature, pressure and dioxygen sensing, as well as in photocatalysis and photodynamic therapy.^21^, ^22^, ^23^, ^24^, ^25^ Replacing one ddpd ligand by tpy slightly increases the excited state lifetime, yet dramatically reduces the quantum yield (Table 1), underlining the positive effect of six‐membered chelate rings.^26^ Photoredox catalytic cycloadditions have been reported using [Cr(phen)_3_]^3+^ derivatives.^27^, ^28^, ^29^, ^30^ Energy transfer to chromium(III) complexes has been investigated using luminescent [Cr(CN)_6_]^3−^, [Cr(ox)_3_]^3−^ or *trans*‐[Cr(CN)_2_(cyclam)]^+^ acceptors in supramolecular architectures and in solid state materials (cyclam=1,4,8,11‐tetraazacyclotetradecane).^31^, ^32^, ^33^ Vice versa, the luminescence of [Cr(alkynyl)_2_(cyclam)]^+^ complexes is quenched by Dexter energy transfer to ferrocene. Furthermore, Cr^III^ complexes have been exploited as sensitizers in molecular lanthanide‐based energy transfer upconversion.^34^

**Table 1:** Luminescence data of pertinent chromium(III) complexes. All data refer to deoxygenated solutions.

complex (solvent)	*τ*/ μs	*Φ*/ %	Ref.
[Cr(phen)_3_]^3+^ (CH_3_CN)	224	0.15	7, 15
[Cr(tpy)_2_]^3+^ (CH_3_CN)	0.14	<0.00089	7, 14
[Cr(ddpd)_2_]^3+^ (H_2_O)	898	11.0	3
[Cr(ddpd)_2_]^3+^ (D_2_O)	1164	14.0	3
[Cr([D_n_]‐ddpd)_2_]^3+^ (CD_3_CN)	2300	30.0	20
[Cr(TAP[9]aneN_3_)]^3+^ (H_2_O)	265	–	16, 17
[Cr(TAP[9]aneN_3_)]^3+^ (D_2_O)	850	–	16, 17
[Cr(*fac*‐Me_5_‐*D*_3*h*_‐tricosaneN_6_)]^3+^ (H_2_O)	235	–	18
[Cr(*fac*‐Me_5_‐*D*_3*h*_‐tricosaneN_6_)]^3+^ (D_2_O)	1500	–	18
[Cr(5‐C≡CH‐bpy)(phen)_2_]^3+^ (CH_3_CN)	259	–	15
[Cr(ddpd)(tpy)]^3+^ (CH_3_CN)	1000	0.06	26

All described and conceivable future applications in luminescence, energy and electron transfer would profit from increased quantum yields and lifetimes. Decisive factors elucidated so far comprise i) a strong ligand field to shift the detrimental ligand field states (^4^T_2g_ in octahedral symmetry) to higher energy^4^, ^35^ and ii) the elimination of high energy XH oscillators from the vicinity of the metal center, for example, by selective deuteration, to reduce non‐radiative multiphonon relaxation.^20^, ^36^, ^37^ A further aspect is to reduce excited state distortion, especially large trigonal twists.^16^, ^17^

Similar to the mainly meridionally coordinating tridentate ligand ddpd,^3^, ^6^, ^38^, ^39^, ^40^, ^41^, ^42^, ^43^ the tripodal ligand 1,1,1‐tris(pyrid‐2‐yl)ethane (tpe)^44^ forms 6‐membered chelate rings with nearly 90° bite angles with transition metal complexes.^45^, ^46^ Tetradentate tpe analogues^47^ were successfully employed in several MCl_2_(L) complexes.^48^ However, chromium(III) complexes with tpe, modified tpe or comparable tpm and tpa ligands have, to the best of our knowledge, not yet been reported (tpm=2,2′,2′‐tripyridylmethane, tpa=2,2′,2′‐tripyridylamine).

We surmised that the tpe ligands should exert a strong ligand field in [Cr(tpe)_2_]^3+^. This should place the lowest energy doublet states below the lowest energy quartet excited states in a homoleptic chromium(III) complex leading to phosphorescence. In contrast to *D*_2_‐symmetric *mer*‐[Cr(ddpd)_2_]^3+^ and *D*_3_‐symmetric [Cr(N∩N)_3_]^3+^ metal complexes, [M(tpe)_2_]^3+^ complexes feature an inversion center. According to Laporte's rule for dd transitions in centrosymmetric complexes, the inversion center should affect the absorption and emission properties.^49^ Furthermore, tpe could be susceptible to ligand‐based redox chemistry (ligand non‐innocence) similar to ^t^bpy, tpy or ^Me^PDP^2−^,^1^, ^2^ contrasting ddpd as a redox‐innocent spectator ligand.^6^

In this study, we exploit the complex [Cr(tpe)_2_]^3+^ with an Earth‐abundant metal ion as a potential substitute for the classical, precious metal containing chromophore [Ru(bpy)_3_]^2+^ in luminescence, as well as in photoinduced energy and electron transfer reactions. Single crystal X‐ray diffraction,^50^, ^51^, ^52^, ^53^, ^54^ NIR luminescence quantum yields^55^ and lifetimes, variable temperature luminescence and step‐scan FT‐IR spectroscopy,^56^, ^57^, ^58^ electrochemistry and spectroelectrochemistry, Stern–Volmer analyses as well as quantum chemical calculations^59^, ^60^, ^61^, ^62^, ^63^, ^64^, ^65^, ^66^, ^67^, ^68^, ^69^ confirm the proposed design guidelines.

## Results and Discussion

### Synthesis and Characterization

**Scheme 2:**
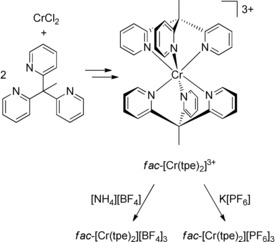
Preparation of the homoleptic chromium(III) complex salts *fac*‐[Cr(tpe)_2_][BF_4_]_3_ and *fac*‐[Cr(tpe)_2_][PF_6_]_3_.

The tripodal pyridine ligand tpe^44^ has been prepared from 2‐ethylpyridine and 2‐fluoropyridine according to a reported procedure.^45^ Treatment of CrCl_2_ with two equivalents of tpe results in complexation and oxidation to Cr^III^. Counterion exchange gives the faint yellow and green complexes *fac*‐[Cr(tpe)_2_][BF_4_]_3_ and *fac*‐[Cr(tpe)_2_][PF_6_]_3_, respectively (Scheme 2). The salts were characterized by mass spectrometry, IR spectroscopy, magnetic susceptibility measurements and elemental analyses. The data support the composition, the high symmetry and the quartet electronic ground state (see Supporting Information, Figures S1–S5).^70^

### Structures in the Solid State and Ground State Quantum Chemical Calculations

**Figure 1:**
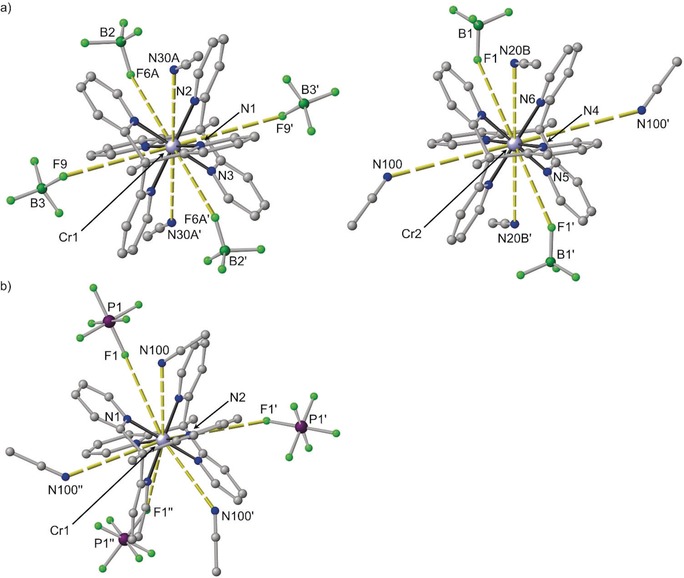
Molecular structures of the cations of a) [Cr(tpe)_2_][BF_4_]_3_×3 CH_3_CN with two independent cations and b) [Cr(tpe)_2_][PF_6_]_3_×3 CH_3_CN including the second coordination sphere of solvents and counterions. View approximately along the molecular threefold axes. Plots of the cations with thermal ellipsoids are depicted in Figure S6.^89^

Single crystals of [Cr(tpe)_2_][BF_4_]_3_×3 CH_3_CN and [Cr(tpe)_2_][PF_6_]_3_×3 CH_3_CN were obtained by diffusion of diethyl ether into acetonitrile solutions of the respective salts (Figure 1). The [BF_4_]^−^ salt crystallizes in the centrosymmetric triclinic space group *P* $\bar{1}$ with two independent trications in the asymmetric unit. Both trications possess crystallographically imposed inversion symmetry. The [PF_6_]^−^ salt crystallizes in the non‐centrosymmetric space group *R*3. The trication possesses crystallographically imposed threefold symmetry without inversion symmetry. The metrical data of the two independent tpe ligands are very similar (Table S1). In all three crystallographically characterized trications, the point symmetry of the [CrN_6_] coordination sphere is close to *O*_h_. The reported d^6^ low spin complexes [Fe(tpe)_2_][ClO_4_]_2_ and [Co(tpe)_2_][ClO_4_]_3_ exhibit a highly symmetric [MN_6_] coordination sphere as well.^45^

Counterions and acetonitrile molecules occupy the six pockets spanned by the pyridyl rings of the tripodal ligands. This second coordination sphere consists of 2 CH_3_CN/4 [BF_4_]^−^, 4 CH_3_CN/2 [BF_4_]^−^ or 3 CH_3_CN/3 [PF_6_]^−^ molecules with Cr**⋅⋅⋅**N/Cr**⋅⋅⋅**F distances ranging from 4.42 to 5.20 Å (Table S1). The low‐spin cobalt(III) salt [Co(tpe)_2_][ClO_4_]_3_×4 CH_3_CN displays a fully analogous 2 CH_3_CN/4 [ClO_4_]^−^ environment with a Co**⋅⋅⋅**N(solvent) distance of 4.76 Å and Co**⋅⋅⋅**O(counterion) distances of 4.20 and 4.50 Å, respectively.^45^ In the monohydrate [Co(tpe)_2_][ClO_4_]_3_×H_2_O, all pockets are filled exclusively with perchlorate counterions with Co**⋅⋅⋅**O distances between 4.47 and 5.09 Å.^45^ The shortest Co**⋅⋅⋅**O(water) distance amounts to 6.49 Å. This is significantly larger than the Cr**⋅⋅⋅**N distances to CH_3_CN. Dynamic solvation and ion pair formation of [M(tpe)_2_]^3+^ cations with variable occupation of the pockets by solvent molecules, especially the sterically less demanding CH_3_CN molecule, and small counterions is expected in solution.^71^, ^72^, ^73^ Furthermore, the terminal methyl groups of the tpe ligands form short H**⋅⋅⋅**F−BF_3_ and H**⋅⋅⋅**F−PF_5_ contacts to the counterions with H**⋅⋅⋅**F distances around 2.5–2.9 Å.

The DFT optimized geometry of [Cr(tpe)_2_]^3+^ at the CPCM(CH_3_CN)‐RIJCOSX‐B3LYP‐D3BJ‐ZORA/def2‐TZVPP level of theory agrees very well with the experimental data (Table S1). The calculated Mulliken α spin density at chromium amounts to ≈3.22 electrons (Figure S7 a). Spin polarization (σ pathway) imposes some β spin density to all six nitrogen donor atoms (Figure S7 a; −0.08) and increases the α spin density at chromium above three. This is in accordance with a (t_2g_)^3^ electron configuration of the chromium(III) ion and the magnetic data (Figure S5). Furthermore, the spin density is consistent with that obtained for [Cr(tpy)_2_]^3+^, [Cr(bpy)_3_]^3+^ and [Cr(MePDP)_2_]^1−^ with 3.21, 3.27 and 3.21 α electrons at the chromium centers, respectively,^1^, ^2^ at the B3LYP/def2‐TZVP level of theory.

### Optical Properties

**Figure 2:**
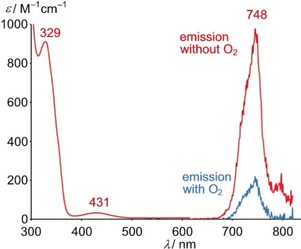
Absorption and emission spectra of [Cr(tpe)_2_][BF_4_]_3_ in D_2_O/DClO_4_ at room temperature (*λ*_exc_=428 nm; 9.0 μl DClO_4_(68 %) mL^−1^ D_2_O) under inert (red) and air‐saturated conditions (blue).

The faint yellow solution of [Cr(tpe)_2_]^3+^ features absorption bands at 329 and 431 nm, irrespective of the counterion ([BF_4_]^−^, [PF_6_]^−^) and the solvent (H_2_O, CH_3_CN) (Figure 2). The weak band at 431 nm (*ϵ*=30 m ^−1^ cm^−1^) corresponds to the strongly parity‐forbidden ^4^A_2g_→^4^T_2g_ ligand field transitions (symmetry labels according to idealized *O*_h_ symmetry). Time‐dependent DFT calculations on the geometry optimized [Cr(tpe)_2_]^3+^ ion (Table S1, quartet state) support this assignment. The spin‐allowed ligand field transitions were calculated at 378, 379 and 394 nm and hence suggest a splitting of the ^4^T_2g_ level (*O*_h_ symmetry) in ^4^E_g_ and ^4^A_1g_ levels by ca. 1000 cm^−1^ due to the actual lower *D*_3*d*_ symmetry. Yet, the inversion center is preserved. In the following discussions, the symmetry labels of the *D*_3*d*_ point group are employed for [Cr(tpe)_2_]^3+^. Due to Laporte's rule, the calculated oscillator strengths are very small (1.4–1.5×10^−8^ each; Figure S7). The analogous absorption band of the comparable centrosymmetric bis(hydrotris(1‐pyrazolyl)borate)chromium(III) complex [Cr(HBpz_3_)_2_]^3+^ is of similar intensity (456 nm; *ϵ*=35 m ^−1^ cm^−1^),^74^ while that of [Cr(ddpd)_2_]^3+^ at 435 nm is more intense by two orders of magnitude due to the lack of the inversion center in [Cr(ddpd)_2_]^3+^.^3^ The “octahedral” ligand field splitting Δ_o_=23 200 cm^−1^ of [Cr(tpe)_2_]^3+^ (corresponding to the center of the quartet absorption band) is in the same range as that of bpy and ddpd chromium(III) complexes, yet larger than that of [Cr(tpy)_2_]^3+^ due to the unfavorable metal‐ligand orbital overlap of the latter.

Excitation of the ligand field states (^4^A_1g_(*D*_3*d*_) and ^4^E_g_(*D*_3*d*_)) is followed by intersystem crossing (ISC) to the doublet manifold, leading to an asymmetric emission band centered at 748 nm (Figure 2). This doublet emission band is relatively broad (FWHM 785 cm^−1^) and appears at higher energy than the corresponding band of [Cr(ddpd)_2_]^3+^ with sharp bands at 775 and 738 nm, a separation typical for the lowest‐energy doublet states of octahedral chromium(III) complexes.^3^, ^75^

**Figure 3:**
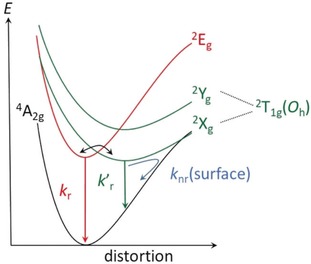
Schematic suggested potential energy curve diagram of [Cr(tpe)_2_]^3+^; term symbols refer to *D*_3*d*_ symmetry, yet with an unknown ordering of ^2^A_2g_(*D*_3*d*_) and ^2^E_g_(*D*_3*d*_) which are denoted arbitrarily as ^2^X_g_(*D*_3*d*_)/^2^Y_g_(*D*_3*d*_).

The *D*_3*d*_ symmetry in [Cr(tpe)_2_]^3+^ splits the ^2^T_1g_(*O*_h_) excited state into ^2^A_2g_(*D*_3*d*_) and ^2^E_g_(*D*_3*d*_) states, while the ^2^E_g_(*O*_h_) level remains degenerate (^2^E_g_(*D*_3*d*_)) (Figure 3). It is conceivable that the lowest doublet state of [Cr(tpe)_2_]^3+^ is one of the split ^2^T_1g_(*O*_h_) states to which electron configurations with two electrons paired in a d(π) orbital, a half‐filled d(π) orbital and an unoccupied d(π) orbital also contribute. This is not the case for ^2^E_g_ in the octahedral limit, a key qualitative difference in electronic structure. Configurations are strongly mixed due to the energetic proximity of the states arising from the ^2^G free ion term and to interaction with ^2^E_g_ and ^2^T_1g_ states arising from higher energy doublet terms of the chromium(III) ion. Consequently, individual configurations cannot be assigned to a single excited state. As the ordering of the ^2^A_2g_(*D*_3*d*_) and ^2^E_g_(*D*_3*d*_) states in [Cr(tpe)_2_]^3+^ cannot be determined from the luminescence spectra, we denote the lowest energy doublet state as ^2^X_g_(*D*_3*d*_) and the higher one as ^2^Y_g_(*D*_3*d*_). Both geometries should be slightly distorted due to the shift in electron density within the d(π) orbitals with respect to the ground state. In the ^2^E_g_ state (derived from ^2^E_g_(*O*_h_)) all three d(π) orbitals are singly occupied and the geometry is very similar to that of the ground state (Figure 3). We will present arguments for this assignment of the doublet levels in the following discussion.

### Excited State Properties

The photoluminescence quantum yield of *Φ*=3.2 % at room temperature in deaerated H_2_O is among the highest reported for chromium(III) complexes, but lower than that of [Cr(ddpd)_2_]^3+^.^3^ The quantum yield of [Cr(tpe)_2_]^3+^ increases in 0.1 m NaClO_4_ (*Φ*=4.2 % in H_2_O/NaClO_4_) or 0.1 m HClO_4_ (*Φ*=5.4 % in H_2_O/HClO_4_). Presumably, the perchlorate and the acid protect the complex, for example, from solvent molecules (cf. the microenvironment of [M(tpe)_2_]^*n*+^ complexes in the solid state).^45^ Consequently, all following luminescence measurements in solution were performed in the presence of perchloric acid (9.5 μl HClO_4_ (70 % in H_2_O) mL^−1^ solvent; 9.0 μl DClO_4_ (68 % in D_2_O) mL^−1^ solvent).

The lifetime *τ*=2800 μs in deaerated H_2_O/HClO_4_ is extremely long and even surpasses that of [Cr(ddpd)_2_]^3+^.^3^ In deaerated D_2_O/DClO_4_, the quantum yield increases to *Φ*=8.2 % and the luminescence lifetime to a record value of *τ*=4500 μs. The photophysical key numbers are similar in deaerated acetonitrile/perchloric acid, which might be an effect of the presence of water in the employed perchloric acid (Table S2). Radiative decay (*k*_r_) is governed by the spin and parity selection rules while the non‐radiative excited state decay (*k*_nr_) of Cr^III^ complexes can occur through back‐intersystem crossing (bISC) to the quartet states (*k*_nr_(bISC)), through surface crossing of distorted excited doublet states with the ground state (*k*_nr_(surface)), through multiphonon relaxation (*k*_nr_(XH)),^20^, ^36^, ^37^ through electronic energy transfer to energy acceptors (*k*_nr_(EnT) and through electron transfer from electron donors (*k*_nr_(ET)).^4^ These decay pathways will be considered in the following to explain the high lifetimes of [Cr(tpe)_2_]^3+^ and to suggest possible applications.

**Figure 4:**
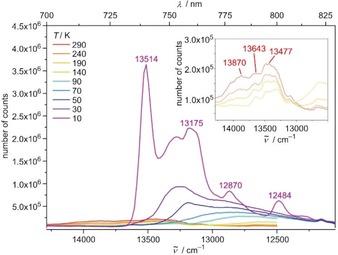
Emission spectra of [Cr(tpe)_2_][BF_4_]_3_ as KBr disk in the temperature range 10–290 K with *λ*_exc_=420 nm. The inset shows a zoom into the spectra in the temperature range 140–290 K.

Due to the inversion center in [Cr(tpe)_2_]^3+^, the luminescence is strictly Laporte‐forbidden. In addition, it is spin‐forbidden as for all chromium(III) complexes with sufficiently strong ligand fields. In fact, the radiative rate constant *k*_r_ of [Cr(tpe)_2_]^3+^ is very small (*τ*_r_=*τ*/ *Φ*=42.9–61.9 ms; *k*_r_=23‐18 s^−1^; Table S2). This is in good agreement with the reported small radiative rate constant *k*_r_=25 s^−1^ (*τ*_r_=40 ms) of the centrosymmetric [Cr(CN)_6_]^3−^ ion.^76^ Vibronic coupling (vibrations of ungerade symmetry) is required to enable this electronic transition in [Cr(tpe)_2_]^3+^. A broad emission band at room temperature has also been observed for the centrosymmetric d^3^ manganese(IV) complex [Mn(PhB(Meim)_3_)_2_]^2+^ with approximate *D*_3*d*_ symmetry ([PhB(MeIm)_3_]^−^=phenyltris(3‐methylimidazol‐2‐yl)borate anion). Its band width (800–2000 cm^−1^; 85–300 K; solid state) has been ascribed to the required vibronic origins involving ungerade parity vibrational modes in centrosymmetric transition metal complexes.^77^, ^78^ On the other hand, complexes lacking an inversion center such as [Cr(bpy)_3_]^3+^ and [Cr(ddpd)_2_]^3+^ feature more symmetric emission bands.^6^ The electronic origin transition of [Cr(tpe)_2_]^3+^ is observed at approximately 13 500 cm^−1^ in the solid‐state emission spectrum at 10 K (Figure 4). It is both spin and parity forbidden, and therefore vibronic origins dominate the observed intensity, both, to higher and lower energy of the origin as temperature increases. The combination of the easily visible origin and the vibrational frequencies of up to 1000 cm^−1^ leads to vibronic origins that cause the relatively broad luminescence band.

Splittings of the asymmetric emission band of [Cr(tpe)_2_]^3+^ at room temperature in the solid state and in solution are discernible (Figures S14–S15; splittings in solution amount to approximately 372 and 346 cm^−1^). DFT calculations find Cr−N stretching vibrations with ungerade symmetry in the range of 300–400 cm^−1^. These vibrations could be the enabling modes leading to the asymmetric emission band with fine structure. However, as several Cr−N vibrations are present in this energy range and as electronic states derived from slightly higher doublet states should have a similar energy (Figure 3), the observed splittings cannot be straightforwardly assigned to vibrational progressions and the experimental energy gaps should not be overinterpreted. Clearly, the inversion center broadens the emission band and reduces *k*_r_ at higher temperature. Possible contributions to *k*_nr_, namely *k*_nr_(bISC), *k*_nr_(surface) and *k*_nr_(XH), will be discussed next.

As the ligand field splitting and consequently the approximate energy difference between the ligand field quartet states ^4^A_1g_(*D*_3*d*_)/^4^E_g_(*D*_3*d*_) and the luminescent doublet states are large (≈1.2 eV), thermally activated bISC to re‐populate the quartet states is highly unlikely. Hence, we exclude the bISC pathway from the discussion for the non‐radiative decay.

A further potential thermally activated non‐radiative decay path could be surface crossing of a distorted doublet state ^2^X_g_(*D*_3*d*_)/^2^Y_g_(*D*_3*d*_) with the ground state (*k*_nr_(surface); Figure 3).^4^, ^79^ The ^2^X_g_(*D*_3*d*_)/^2^Y_g_(*D*_3*d*_) potential energy surface should be displaced horizontally relative to the ground state minimum, enabling efficient non‐radiative relaxation pathways. Such a doublet potential well displacement has been suggested before and denoted “pseudo‐Stokes shift” giving rise to a low energy phosphorescence.^79^ Upon cooling crystals of [Cr(tpe)_2_][BF_4_]_3_ to 80 K the broad emission band centered at 745 nm (13 425 cm^−1^) disappears while a new broad, structured band at lower energy (centered at ca. 770 nm/13 000 cm^−1^) grows in (Figure S16). The intensity decrease in the 700 nm to 740 nm range is clearly visible in Figure S16 and is the typical signature of thermally populated electronic or vibrational levels with higher radiative relaxation rates than the lowest‐energy electronic transition. In the title compound, the effect is dramatic due to its exact inversion symmetry and leads to exceptional variations of the spectroscopic patterns. Crystals of [Cr(tpe)_2_][PF_6_]_3_ display a fully analogous overall behavior upon cooling to 80 K, yet with slightly different vibrational fine structure (Figure S17). Even KBr disks of [Cr(tpe)_2_][BF_4_]_3_ show an increasing low‐energy emission band at lower temperature at the expense of a high energy band (Figure 4). At 10 K, the low energy band shows resolved fine structure and the intensity dramatically increases (Figure 4). The increase in emission intensity is compatible with the proposed diminished *k*_nr_(surface) at lower temperature of the “pseudo‐Stokes shifted” ^2^X_g_(*D*_3*d*_) state (Figure 3).^79^

**Figure 5:**
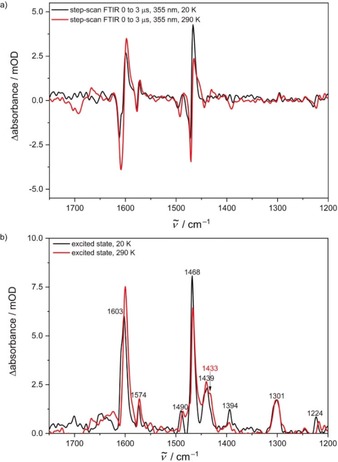
a) Step‐scan FTIR spectra of [Cr(tpe)_2_][BF_4_]_3_ at 290 K (red) and 20 K (black) in a KBr disk 0 to 3 μs after laser excitation at 355 nm and b) IR spectra of the excited state after subtraction of the spectrum of the electronic ground state at 290 K (red) and 20 K (black).

To gain more insight into the geometries of the long‐lived excited states, we subjected KBr disks of [Cr(tpe)_2_][BF_4_]_3_ to time‐resolved step‐scan FTIR spectroscopy^56^, ^57^, ^58^, ^80^ in the energy range of 1750 to 1200 cm^−1^ at 290 and 20 K (Figure 5 a). The negative bands in the difference spectra indicate depopulation of the ground state while positive bands belong to the electronically excited state(s). DFT calculations excellently reproduce the ground state IR spectrum (Figure S18). In the excited state, nearly all IR bands shift to lower energy and a shoulder appears at approximately 1433 cm^−1^ at 290 K (Figure 5 b). The shoulder might appear due to the population of two long‐lived excited states or to the removal of the inversion center in the doublet states. At 20 K, the IR bands in the step‐scan IR spectra sharpen and the shoulder disappears. This might point to a preferred population of a long‐lived excited state at 20 K (^2^X_2_(*D*_3*d*_)) which would match the suggested excited state ordering (Figure 3). From time‐resolved IR data at low and high temperature, biexponential decays are extracted (20 K: 66 μs (82 %); 2.2 μs (18 %); 290 K: 10 μs (65 %); 0.71 μs (35 %), Figure S19).

However, the data of the TCSPC experiments at 290 K in KBr disks are fitted with tri‐ and tetraexponential decay curves (Figures S20–S22). The latter one results in a slightly better description with respect to the residuals. All fits yield two long‐lived components, which are associated with two emissive doublet states at 290 K. The triexponential function shows one time constant in the nanosecond regime, whereby the tetraexponential fit yields a further short‐lived component with a very small contribution equal or less to 3 %. A true photophysical relevance of this fourth component might be questionable. The nanosecond processes may be associated to fluorescence. Static emission and TCSPC investigations on neat films confirm the results from KBr disks and indicate that no matrix effects of KBr are observed (Figure S23).

A biexponential decay is observed in both, the step‐scan and the TCSPC experiments at 20 K (Figure S24–S25). The biexponential decay from TCSPC measurements shows a very small, negligible contribution (2–5 %) for the shorter‐lived component indicating a low population of the energetically higher excited doublet state. The step‐scan data also show a smaller, yet significant contribution (18 %) of the shorter‐lived component at 20 K (compared to 290 K). This comparably high population cannot result from a pure thermalized occupation of the low energy ^2^E_g_/^2^X_g_ states but could result from differently efficient population transfer from the initially excited quartet states to the doublets, for example, to ^2^X_g_/^2^Y_g_ in an approximately 82:18 ratio. The decay of the second component to the ground state contains a prominent non‐radiative pathway based on the different IR and TCSPC results. Some of the non‐radiative pathways might be assigned to surface crossing and multiphonon relaxation (*k*_nr_(XH)).

**Scheme 3:**
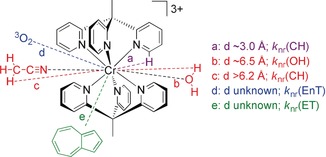
Possible non‐radiative decay pathways of [Cr(tpe)_2_]^3+^: multiphonon relaxation by ligand CH modes (a: in purple), by water OH modes (b: in red), by acetonitrile CH modes (c: in red); Dexter energy transfer to ^3^O_2_ (d: in blue) and electron transfer from azulene Az (e: in green). Distances to CH_3_CN and H_2_O estimated from XRD analyses (see above).

Indeed, multiphonon relaxation typically plays a decisive role in non‐radiative excited state decay of NIR emitters.^4^, ^20^, ^36^, ^37^ The fourth vibrational overtone of aromatic CH oscillators of pyridyl ligands (14 065 cm^−1^)^22^ falls within the emission band envelope of the room temperature emission band of [Cr(tpe)_2_]^3+^ (Figure S15). This results in an appreciable spectral overlap integral, hence promoting non‐radiative deactivation by nearby CH oscillators (Scheme 3; pyridyl CH group with a Cr**⋅⋅⋅**H distance of *d*≈3.0 Å). [Cr(tpe)_2_]^3+^ features six nearby CH oscillators (Scheme 3), while [Cr(ddpd)_2_]^3+^ only provides four of them (Scheme 1). This further increases *k*_nr_(XH) of [Cr(tpe)_2_]^3+^ with respect to that of [Cr(ddpd)_2_]^3+^. However, at 10 K, the emission of the distorted ^2^X_2_(*D*_3*d*_) state of [Cr(tpe)_2_]^3+^ shifts to lower energy (Figure 4), significantly reducing the spectral overlap integral and consequently enhancing the quantum yield.

Similar to CH oscillators of the ligand, OH oscillators of the solvent quench the phosphorescence (Table S2; Scheme 3; Cr**⋅⋅⋅**O distance *d*≈6.5 Å). This solvent‐promoted non‐radiative decay is less likely in heavy water due to the required higher overtone of the OD vibration. This increases quantum yield and lifetime by 1.5–1.6. Changing the medium from H_2_O/HClO_4_ to CH_3_CN/HClO_4_ (and D_2_O/DClO_4_ to CD_3_CN/DClO_4_) barely affects the photophysical data (Table S2). This might be an effect of the water/perchlorate present with the perchloric acid providing a similar microenvironment around the complex under all conditions (cf. Figure 1). This assumption is substantiated by estimating the expected effect of CH_3_CN deuteration according to the theory of multiphonon relaxation.^36^, ^37^ Determination of CH/CD overtone and combination mode energies and extinction coefficients of CH_3_CN/CD_3_CN allowed calculating the expected spectral overlap integrals with the luminescence band. In fact, *k*_nr_(CD) should be smaller than *k*_nr_(CH) by more than three orders of magnitude (for details see Figures S26–S33), provided this pathway would play a significant role. This is clearly not the case and hence, *k*_nr_(CH) through acetonitrile CH modes is not particularly relevant under these acidic/wet conditions.

We suggest that a close to octahedral symmetry of the [CrN_6_] polyhedron with N‐Cr‐N angles of ≈90°/≈180° is beneficial for high *Φ* as the resulting strong ligand field shifts the quartet states to higher energy (small *k*_nr_(bISC)). Yet, an inversion center of the entire complex (e.g. *O*_h_, *D*_4*h*_, *D*_3*d*_ point groups), including the π planes of the pyridine rings, reduces *k*_r_ and hence, the quantum yield. Shielding of the complex from solvent XH modes decreases *k*_nr_(XH). Both, small *k*_r_ and *k*_nr_ lead to the exceptionally high lifetime *τ* of [Cr(tpe)_2_]^3+^. The long lifetime should favor bimolecular reactions with substrates, namely energy transfer (*k*(EnT)) and electron transfer (*k*(ET)) (Scheme 3).

In acidic, air‐saturated water, oxygen quenches the spin‐flip emission of [Cr(tpe)_2_]^3+^ with *τ*(Ar) : *τ*(O_2_) ratios of 2.5 (H_2_O) and 2.0 (D_2_O) (Scheme 3, path d; *k*_nr_(EnT); Table S2). Similar values are found in acetonitrile/acid mixtures. Obviously, the typical Dexter energy transfer pathway^81^ of the doublet state(s) to ^3^O_2_ forming ^1^O_2_ is viable for [Cr(tpe)_2_]^3+^ (Scheme 3, blue quenching pathway).^26^ The very high lifetime favors this pathway and enables applications of [Cr(tpe)_2_]^3+^ in photo‐induced energy transfer reactions.

### Ground State and Excited State Redox Properties

[Cr(tpe)_2_]^3+^ exhibits two reversible one‐electron reduction waves at *E*_1/2_=−0.88 and −1.54 V and a quasireversible reduction peak at *E*_p_=−2.49 V (Figures S34–S35). [Cr(ddpd)_2_]^3+^ is reduced to the corresponding labile d^4^ chromium(II) complex at *E*_1/2_=−1.11 V and irreversibly at the ddpd ligand at −1.94 V vs. FcH/FcH^+^.^3^, ^6^ On the other hand, [Cr(tpy)_2_]^3+^ (*E*_1/2_=−0.53, −0.95, −1.45, −2.37 V)^2^ and [Cr(^t^bpy)_3_]^3+^ (*E*_1/2_=−0.63, −1.15, −1.72, −2.34, −2.67, −2.90 V)1a exhibit several reversible one‐electron reduction steps, which have all been assigned to ligand centered reductions yielding the corresponding radical anions and dianions coordinated to Cr^3+^. [Cr(^t^bpy)_3_]^*n*+^ and [Cr(tpy)_2_]^*n*+^ (*n*=2, 1) exhibit characteristic intense absorption bands in the red to near‐infrared spectral region. On the basis of their intensity (*ϵ* in the order of several 1000 m ^−1^ cm^−1^) and time‐dependent DFT calculations, these bands have been assigned to π‐π* transitions of the ^t^bpy^⋅−^/tpy^.−^ radical anions.^1^

In order to determine the site of reduction, [Cr(tpe)_2_]^3+^ was subjected to reductive electrolysis inside a transparent UV/Vis/NIR cell under the in situ conditions of spectroelectrochemistry.^82^ Exemplary spectra are displayed in Figure S36. Most notably, intense bands in the red to near‐infrared spectral region grow in. The intensities of these bands with *ϵ*>1000 m ^−1^ cm^−1^ are clearly incompatible with a chromium(II) or chromium(I) complex description as these complexes should only display weak Laporte‐forbidden transitions. The observed intense bands resemble those of [Cr(bpy)_3_]^*n*+^ and [Cr(tpy)_2_]^*n*+^ (*n*=2, 1) with coordinated ligand radical anions.^1^ Consequently, we assign these bands to π‐π* transitions of coordinated tpe^⋅−^ radical ligands. TD‐DFT calculations on geometry optimized [Cr(tpe)_2_]^2+^ (*S=*1) and [Cr(tpe)_2_]^+^ (*S*=1/2 ) cations find charge transfer absorption bands in these spectral regions as well (Figure S37). The good agreement substantiates the *S=*1 and *S*=1/2 ground state of [Cr(tpe)_2_]^2+^ and [Cr(tpe)_2_]^+^, respectively. This fully agrees with previously reported complexes of chromium(III) and pyridine radical ligands.^1^, ^2^

**Figure 6:**
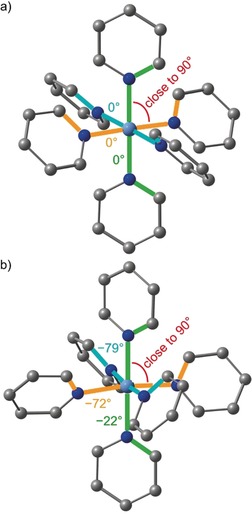
Close to octahedral symmetry of the [CrN_6_] cores and the different orientations of the pyridine ligands in a) [Cr(tpe)_2_]^3+^ and b) [Cr(ddpd)_2_]^3+^ complexes. The bridging atoms and hydrogen atoms of the ligands are omitted and the N‐Cr‐N (in red) and C‐N‐N‐C angles (in blue, green and orange) given in deg.

**Scheme 4:**
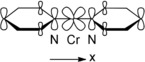
Relevant spin‐carrying ligand π* and metal d orbitals along the *x* axis in [Cr(tpe)_2_]^*n*+^ (*n*=1, 2); analogous combinations are formed along the *y* and *z* directions.

DFT calculations of the respective di‐ and monocations are consistent with a description as chromium(III) ions coordinated by radical ligands, although the Mulliken α spin density at Cr corresponds to less than three unpaired electrons (Figure S7). This suggests strong interactions between the 3d(π) orbitals and the π system of the ligands. The β spin density distribution cannot be assigned to individual tpe ligands, but is rather delocalized over two *trans*‐coordinated pyridines of different tpe ligands featuring a coplanar orientation (Figure S7 b and S7 c; Scheme 4). This contrasts with the behavior of the radical ligands in [Cr(^t^bpy)_3_]^*n*+^, [Cr(tpy)_2_]^*n*+^ and [Cr(^Me^PDP)_2_]^2−^ coordinated in an orthogonal arrangement of the ligand planes. The *trans*‐coordinated pyridine pairs in [Cr(tpe)_2_]^*n*+^ are perfectly co‐planar and aligned with the chromium d(π) orbitals (Scheme 4; Figure 6 a). This generates three molecular orbitals, each comprised of two pyridine π* orbitals and a symmetry‐corresponding d(π) orbital of the metal (Scheme 4; py‐Cr‐py). In [Cr(tpe)_2_]^2+^ (triplet state), three electrons occupy the metal d(π) orbitals with α spins and the fourth electron occupies a molecular orbital composed of two π* orbitals of *trans*‐coordinated pyridines with a β spin (antiferromagnetic coupling to one α spin in the corresponding d(π) orbital; Figure S7 b).

In the monocation [Cr(tpe)_2_]^+^ (doublet state), three electrons occupy the metal d(π) orbitals with α spins. The two β electrons are symmetrically delocalized over all six pyridines with a net antiferromagnetic coupling to two α spins in the chromium d(π) orbitals (Figure S7 c). A delocalized model has also been calculated for [Cr(bpy)_3_]^+^ featuring three identical β spin‐carrying bpy ligands (point group *D*_3_) although with a larger spin density at the metal center.1a That delocalized descriptions are favored could be an intrinsic bias of the B3LYP functional^1^ or due to the lack of charge‐localizing and symmetry‐breaking counterions in the computational model. The suitable description of the electronic structures of [Cr(bpy)_3_]^*n*+^, [Cr(tpy)_2_]^*n*+^ and [Cr(tpe)_2_]^*n*+^ (*n*=2, 1) as chromium(III) ions coordinated by radical ligands is, however, demonstrated in all cases and consistent with UV/Vis/NIR spectroscopic data. Reduction of pyridines coordinated to Cr^III^ becomes more difficult in the series tpy, ^t^bpy,^1^ and tpe and hence, the latter case approaches other limiting electronic situation [Cr^II^L_3_]^2+^ with an essentially metal centered reduction as realized in [Cr^II^(ddpd)_2_]^2+^.^3^, ^6^ This sequence can be attributed to the type and energy of the π* system hosting the additional electron, namely Cr(**py‐py‐py**), Cr(**py‐py**), and [**py**‐Cr‐**py**] (Scheme 4) for [Cr(tpy)_2_]^2+^, [Cr(bpy)_3_]^2+^, and [Cr(tpe)_2_]^2+^, respectively.

With the doublet state energy of [Cr(tpe)_2_]^3+^ of ca. 1.75 eV at room temperature (from the 0‐0 energy of the emission band fit, Figure S15) and the redox potential of the [Cr(tpe)_2_]^3+/2+^ couple, the excited state reduction potential amounts to +0.87 V vs. FcH/FcH^+^ (+1.25 V vs. SCE^83^). This exceeds the potential of commonly employed photoredox catalysts [Ru(bpy)_3_]^2+^ (+0.77 V vs. SCE^84^) and *fac*‐Ir(ppy)_3_ (+0.31 V vs. SCE;^27^ ppy=anion of 2‐phenylpyridine), yet is smaller than that of the strongest chromium(III) derived photooxidants ([Cr(dmcbpy)_3_]^3+^: +1.84 V vs. SCE; dmcbpy=4,4′‐di(methylcarboxyl)‐2,2′‐bipyridine;^27^ [Cr(ttpy)_2_]^3+^: +1.44 V vs. SCE; ttpy=4′‐(*p*‐tolyl)‐2,2′:6,2′′‐terpyridine).^85^ In terms of excited state lifetime, [Cr(tpe)_2_]^3+^ surpasses all these sensitizers by orders of magnitude (*τ*([Ru(bpy)_3_]^2+^)=1.1 μs; *τ*(*fac*‐Ir(ppy)_3_)=1.9 μs; *τ*([Cr(dmcbpy)_3_]^3+^)=7.7 μs; *τ*([Cr(ttpy)_2_]^3+^)=0.27 μs).^27^, ^85^

To probe the photoredox chemistry of [Cr(tpe)_2_]^3+^, azulene (Az) was employed as substrate. The triplet energy of Az (1.74 eV)^86^ is close to the doublet state energy of [Cr(tpe)_2_]^3+^, hence energy transfer to Az is less favorable. On the other hand, Az can be oxidized to its radical cation Az^⋅+^ at 0.50 V vs. FcH/FcH^+^ (0.88 V vs. SCE)^87^ which is significantly lower than the excited state redox potential of [Cr(tpe)_2_]^3+^, proving enough driving force for the electron transfer. Az quenches the luminescence of [Cr(tpe)_2_]^3+^ with a Stern–Volmer constant *K*_SV_=41.7×10^3^ m ^−1^ (Figure S38). The efficient luminescence quenching by Az supports photoinduced electron transfer from Az to ^2^[Cr(tpe)_2_]^3+^ (Scheme 3, *k*_nr_(ET)), that is, to the redox orbital formed by tpe ligands and the chromium ion (Scheme 4). Additionally, tri(*n*‐butyl)amine (*E* ^p^=0.38 V vs. FcH/FcH^+^)^24^ quenches the luminescence of [Cr(tpe)_2_]^3+^ forming [Cr(tpe)_2_]^2+^ according to UV/Vis/NIR spectroscopy (Figure S39). Further substrates suitable for activation by [Cr(tpe)_2_]^3+^ will be reported in future studies.

## Conclusion

The centrosymmetric complex [Cr(tpe)_2_]^3+^ is highly luminescent at room temperature in D_2_O/DClO_4_ (*Φ*=8.2 %) due to a large ligand field splitting (23 200 cm^−1^). The emission (13 370 cm^−1^) is strongly Laporte‐forbidden, leading to an unprecedentedly high luminescence lifetime (4500 μs). The most relevant non‐radiative pathways of the luminescent states are surface crossing with the ground state and multiphonon relaxation through nearby CH oscillators of the tpe ligand as well as through solvent modes (OH in water). Energy transfer from the excited state to triplet oxygen is feasible as well.

The coplanar orientation of *trans*‐coordinated pyridine donors in [Cr(tpe)_2_]^3+^ enables ligand‐based reduction processes. The additional electrons occupy π* orbitals delocalized over two tpe ligands (and some Cr) in [Cr(tpe)_2_]^2+/+^ ions. These coordinated π radicals show strong NIR absorption bands. Consequently, a conjugated oligopyridine ligand, as found in the electron transfer series [Cr(^t^bpy)_3_]^*n*+^ and [Cr(tpy)_2_]^*n*+^, is not required for ligand‐based redox‐chemistry.

The very long excited state lifetime and ligand‐centered reduction of [Cr(tpe)_2_]^3+^ enable both, energy and electron transfer processes with suitable substrates such as oxygen, azulene and tri(*n*‐butyl)amine. This excited state reactivity paves the way for employing this specific [CrN_6_] chromophore architecture in energy transfer schemes such as singlet oxygen formation,^26^ triplet sensitizing^88^ or lanthanide‐based energy transfer upconversion34b as well as in photoredox catalysis.^27^, ^28^, ^29^, ^30^ Work in these directions is currently in progress in our laboratories.

## Conflict of interest

The authors declare no conflict of interest.

## Supporting information

As a service to our authors and readers, this journal provides supporting information supplied by the authors. Such materials are peer reviewed and may be re‐organized for online delivery, but are not copy‐edited or typeset. Technical support issues arising from supporting information (other than missing files) should be addressed to the authors.

Supplementary
