# Supplementary material for: Luminescence and Light‐Driven Energy and Electron Transfer from an Exceptionally Long‐Lived Excited State of a Non‐Innocent Chromium(III) Complex
Source: Angew Chem Int Ed Engl. 2019 Oct 31;58(50):18075–85. doi: 10.1002/anie.201909325 (PMC6916301; doi:10.1002/anie.201909325)
Supplement: Supplementary file 1 — Supplementary [file ANIE-58-18075-s001.pdf]

Angewandte Chemie
Eine Zeitschrift der Gesellschaft Deutscher Chemiker

Supporting Information

Luminescence and Light-Driven Energy and Electron Transfer from an Exceptionally Long-Lived Excited State of a Non-Innocent Chromium(III) Complex

*Steffen Treiling, Cui Wang, Christoph Förster, Florian Reichenauer, Jens Kalmbach, Pit Boden, Joe P. Harris, Luca M. Carrella, Eva Rentschler, Ute Resch-Genger, Christian Reber, Michael Seitz, Markus Gerhards, and Katja Heinze**

anie_201909325_sm_miscellaneous_information.pdf

Author Contributions

The manuscript was written through contributions of all authors. ST synthesized the tpe ligand and the tpe complexes, performed ground state IR, MS, CV and SEC studies as well as most DFT calculations. CF performed further DFT calculations, and solved and refined the X-ray structures, CW and URG performed the quantum yield and lifetime measurements in solution, LC and ER performed and analyzed the magnetic measurements, JK and MS performed the IR overtone spectroscopy and SOI calculations, PB and MG performed and interpreted the temperature dependent transient IR spectra and luminescence experiments in KBr disks and neat films, JPH and CR performed and interpreted low-temperature luminescence experiments in the crystals, FR prepared more material and performed the Stern–Volmer analysis. KH designed the concept, interpreted the data and wrote the manuscript. All authors have given approval to the final version of the manuscript.

Supporting Information

General Procedures. Diethyl ether was distilled over sodium, THF over potassium and acetonitrile over calcium hydride. The ligand tpe was prepared similar to a reported procedure.[S1] NMR spectroscopic and mass spectrometric data match the literature values. A glovebox (UniLab/MBraun, Ar 4.8, O_2 < 100 ppm, H_2O < 1 ppm) was used for storage and weighing of sensitive compounds. Reagents were received from usual suppliers (ABCR, Acros Organics, Alfa Aesar, Fischer Scientific, Fluka and Sigma Aldrich). NMR spectra of tpe were recorded on a Bruker Avance DRX 400 spectrometer at 400.31 MHz (^{1}H). All resonances are reported in ppm versus the solvent signal as internal standard [$CDCl_3$ (^{1}H: δ = 7.26)].[S2] IR spectra were recorded with a Bruker Alpha FTIR spectrometer with ATR unit containing a diamond crystal. ESI^+ mass spectra were recorded on a Micromass Q-TOF-Ultima spectrometer. DC magnetic studies were performed with a Quantum Design MPMS-XL-7 SQUID magnetometer on powdered microcrystalline samples. Experimental susceptibility data were corrected for the underlying diamagnetism using Pascal's constants. The temperature dependent magnetic contribution of the holder and of the embedding eicosane matrix was experimentally determined and subtracted from the measured susceptibility data. Variable temperature susceptibility data were collected in a temperature range of 6 – 300 K under an applied field of 0.1 Tesla. Electrochemical experiments were carried out on a BioLogic SP-50 voltammetric analyzer using platinum wires as counter and working electrodes and a 0.01 M Ag/Ag[NO_3] electrode as reference electrode. Cyclic voltammetry and square wave measurements were carried out at scan rates of 50–200 mV s^{-1} using 0.1 M [N^nBu_4][BF_4] in CH_3CN as supporting electrolyte. Potentials are referenced against the ferrocene/ferrocenium couple. Spectroelectrochemical experiments were performed using a Specac omni-cell liquid transmission cell with CaF_2 windows equipped with a Pt gauze working electrode, a Pt gauze counter electrode and a Ag wire as pseudo reference electrode, melt-sealed in a polyethylene spacer (approximate path length 0.5 mm) in 10^{-5} M solutions in CH_3CN, containing 0.1 M [N^nBu_4][BF_4]. UV/Vis/NIR spectra were recorded on a Varian Cary 5000 spectrometer using 1.0 cm cells. Luminescence emission spectra and decays in solution were reported with a calibrated spectrofluorometer FSP 920 from Edinburgh Instruments. For the measurement of the emission spectra, a continuous xenon lamp was applied as excitation light source, while the time-resolved luminescence measurements were completed with a µs xenon flashlamp and detection in a multi-channel scaling mode. All measurements were performed at magic angle condition (polarization 0° in the excitation and 54.7° in the emission channel). The luminescence decays in solution were analyzed by fitting the obtained decay curves mono-exponentially with the program FAST (Fluorescence Analysis Software Technology, Edinburgh Instruments Ltd.). The luminescence quantum yields in solution were determined using an Ulbricht integrating sphere (Quantaurus-QY C11347-11, Hamamatsu).[S3-S5] Relative uncertainty is estimated to be ±5 %. NIR absorption spectra of CH_3CN and CD_3CN for overtone determination were measured in absorption mode using a JASCO V-770 spectrophotometer (equipped with a long cuvette holder LSE-701). The spectra were recorded in rectangular semimicro cuvettes (Starna, type 28/B/SX/50, path length 5.0 cm). CH_3CN was spectrophotometric grade and CD_3CN was NMR grade (99.8% D). The spectra were corrected for baseline drift with OriginPro 9.0. The component peaks of the spectra were deconvoluted by fitting of the spectra with a series of Gaussian functions (Levenberg-Marquardt on χ^2). All time-resolved FTIR experiments were performed with an FTIR spectrometer Bruker Vertex 80v, operated in the step-scan mode. KBr pellets of [$Cr(tpe)_2$][BF_4]$_3$ (ca. 0.75 mg) were prepared by mixing with dry KBr (ca. 200 mg, stored at 80 °C) and grinding to a homogeneous mixture. The strongest peak in the ground state spectrum showed an absorption of about 0.6 OD with the mentioned concentration. Measurements with cryogenically cooled KBr pellets (20 K and 290 K at the sample) were performed with a closed cycle helium cryostat (ARS Model DE-202A). The cryo cooler was equipped with a

homebuilt pellet holder and CaF_2 windows. A liquid-nitrogen-cooled mercury cadmium telluride (MCT) detector (Kolmar Tech., Model KV100-1-B-7/190) with a rise time of 25 ns, connected to a fast preamplifier and a 14-bit transient recorder board (Spectrum Germany, M3I4142, 400 MS s^{-1}), was used for signal detection and processing. The laser setup includes a Q-switched Nd:YAG laser (Innolas SpitLight Evo I) generating pulses with a band-width of 6 – 9 ns at a repetition rate of 100 Hz. The third harmonic (355 nm) of the Nd:YAG laser was used for sample excitation. The UV pump beam was attenuated to about 2.0 mJ per shot at a diameter of 9 mm. The beam was directed onto the sample and adjusted to have a maximal overlap with the IR beam of the spectrometer. The sample chamber was equipped with anti-reflection-coated germanium filters to prevent the entrance of laser radiation into the detector and interferometer compartments. The time delay between the start of the experiment and the UV laser pulse was controlled with a Stanford Research Systems DG535 delay generator. A total of more than 2000 coadditions were recorded at each interferogram point. The spectral region was limited by undersampling to 988 – 1975 cm^{-1} with a spectral resolution of 4 cm^{-1} resulting in 555 interferogram points. An IR broad band filter (850 – 1750 cm^{-1}) and the CaF_2 windows (no IR transmission $<$ 1000 cm^{-1}) of the cryostat prevented problems when performing a Fourier transformation (*i.e.* no IR intensity outside the measured region should be observed). FTIR ground state spectra were recorded systematically to check for sample degradation. A more detailed description of the step-scan setup is given here.[S6-S8] Temperature dependent emission spectra of KBr disks were recorded with a FluoroMax-2 (Horiba Scientific) device using the described cryostat. Time-correlated single photon counting measurements were performed with a DeltaFlex (Horiba Scientific) instrument at a repetition rate of 10 kHz with a time resolution of 13 or 27 ns, depending on the experiment. Temperature dependent emission spectra of crystals of $[Cr(tpe)_2][BF_4]_3$ and $[Cr(tpe)_2][PF_6]_3$ were recorded with a Renishaw Invia Raman microscope equipped with a Peltier-cooled CCD camera. The excitation source was a 488 nm Argon ion laser. Variable-temperature spectra were obtained by coupling a Linkam cryostat to the microscope with liquid nitrogen used as the coolant. Elemental analyses were conducted by the microanalytical laboratory of the chemical institutes of the University of Mainz.

Caution! Although we have not experienced any problems in handling the perchlorate solutions, all materials should be handled with extreme care.

Crystal structure determinations. Diffusion of diethyl ether into concentrated solutions of $[Cr(tpe)_2][BF_4]_3$ or $[Cr(tpe)_2][PF_6]_3$ in CH_3CN yielded diffraction quality crystals. Intensity data were collected with a STOE IPDS-2T diffractometer and an Oxford cooling system and corrected for absorption and other effects using Mo K_α radiation (λ = 0.71073 Å). The diffraction frames were integrated using the SAINT package, and most were corrected for absorption with MULABS.[S9,S10,S11] The structures were solved by direct methods and refined by the full-matrix method based on F^2 using the SHELXTL software package.[S12,S13] All non-hydrogen atoms were refined anisotropically, while the positions of all hydrogen atoms were generated with appropriate geometric constraints and allowed to ride on their respective parent carbon atoms with fixed isotropic thermal parameters. CCDC 1876389 ($[Cr(tpe)_2][BF_4]_3\times3CH_3CN$) and 1876390 ($[Cr(tpe)_2][PF_6]_3\times3CH_3CN$) contain the supplementary crystallographic data for this paper. These data are provided free of charge by The Cambridge Crystallographic Data Centre.

Crystallographic Data of $[Cr(tpe)_2][BF_4]_3\times3CH_3CN$: $C_{40}H_{39}B_3CrF_{12}N_9$ (958.23); triclinic; $P\bar{1}$; a = 11.3010(8) Å, b = 13.5207(8) Å, c = 15.5147(9) Å, α = 74.575(4)°, β = 76.869(5)°, γ = 73.432(5)°; V = 2150.6(2) Å^3; Z = 2; density, calcd. = 1.480 g cm^{-3}, T = 120(2) K, μ = 0.361 mm^{-1}; F(000) = 978; crystal size 0.600 × 0.310 × 0.187 mm; θ= 1.915 to 28.318 deg.; –15≤h≤15, –17≤k≤18, –20≤l≤20; rfln collected = 39128; rfln unique = 10667 [R(int) = 0.0604]; completeness to θ= 25.242 deg. = 99.9 %; semi empirical

absorption correction from equivalents; max. and min. transmission 1.11561 and 0.88170; data 10667; restraints 300, parameters 861; goodness-of-fit on F^2 = 1.038; final indices [$I > 2\sigma(I)$] R_1 = 0.0556, wR_2 = 0.1342; R indices (all data) R_1 = 0.0871, wR_2 = 0.1546; largest diff. peak and hole 0.454 and –0.557 e Å^{-3}.

Crystallographic Data of [Cr(tpe)$_2$][PF$_6$]$_3$×3CH$_3$CN: $C_{40}H_{39}CrF_{18}N_9P_3$ (1132.71); trigonal; $R3$; $a = b$ = 16.953(2) Å, c = 13.901(3) Å; V = 3459.9(11) Å^3; Z = 3; density, calcd. = 1.631 g cm^{-3}, T = 193(2) K, μ = 0.468 mm^{-1}; F(000) = 1719; crystal size 0.310 × 0.240 × 0.190 mm; θ = 2.403 to 28.293 deg.; $-20 \leq h \leq 19$, $-19 \leq k \leq 22$, $-18 \leq l \leq 18$; rfln collected = 6617; rfln unique = 3811 [R(int) = 0.0213]; completeness to θ = 25.242 deg. = 99.9 %; semi empirical absorption correction from equivalents; max. and min. transmission 1.04896 and 0.95585; data 3811; restraints 1, parameters 217; goodness-of-fit on F^2 = 1.071; final indices [$I > 2\sigma(I)$] R_1 = 0.0334, wR_2 = 0.0838; R indices (all data) R_1 = 0.0378, wR_2 = 0.0865; largest diff. peak and hole 0.375 and –0.195 e Å^{-3}.

Density functional theoretical calculations on the chromium complex cations [Cr(tpe)$_2$]$^{n+}$ were carried out using the ORCA program package (version 4.0.1).[S14] Tight convergence criteria were chosen for all calculations (keywords tightscf and tightopt). All calculations make use of the resolution of identity (Split-RI-J) approach for the Coulomb term in combination with the chain-of-spheres approximation for the exchange term (COSX).[S15,S16] Geometry optimization was performed using the B3LYP functional[S17] in combination with Ahlrichs' split-valence triple-ζ basis set def2-TZVPP for all atoms.[S18,S19] The optimized geometries were confirmed to be local minima on the respective potential energy surface by subsequent numerical frequency analysis (N_{imag} = 0). TD-DFT calculations were performed at the same level of theory. Fifty vertical spin-allowed transitions were calculated. The zero order relativistic approximation was used to describe relativistic effects in all calculations (keyword ZORA).[S20,S21] Grimme's empirical dispersion correction D3(BJ) was employed (keyword D3BJ).[S22,S23] To account for solvent effects, a conductor-like screening model (keyword CPCM) modeling acetonitrile was used in all calculations.[S24] Explicit counterions and/or solvent molecules were neglected.

Synthesis of 1,1,1-tris(pyrid-2-yl)ethane tpe:[S1] 2-Ethylpyridine (2.1 ml, 18.7 mmol) in dry THF (60 ml) were cooled to –78°C. ^{n}Butyl lithium (2.5 M in hexane; 7.5 ml; 18.75 mmol) was added dropwise. After stirring for 50 min and warming to –40°C, 2-fluoropyridine (3.6 g. 37.4 mmol) was added to the red solution with the temperature kept below –30°C. The resulting colorless reaction mixture was warmed to room temperature and then heated under reflux for 12 h. After cooling to room temperature, the solvents were removed under reduced pressure. The resulting solid was dissolved in THF (60 ml) and filtered. The solvent was removed under reduced pressure and the brown raw product was purified by column chromatography (alumina, hexanes/ethyl acetate 2:1, R_f = 0.30) giving tpe as off-white solid (2.0 g, 7.6 mmol, 41 %). $C_{17}H_{15}N_3$ (261.33). ^{1}H NMR (CDCl$_3$): δ = 8.50 (d, $^3J_{HH}$ = 3.5 Hz, 3H, H^4), 7.65 (dt, $^3J_{HH}$ = 7.7 Hz, $^3J_{HH}$ = 1.6 Hz, 3H, H^2), 7.21 (dd, $^3J_{HH}$ = 6.7 Hz, $^3J_{HH}$ = 4.9 Hz, 3H, H^3), 7.11 (d, $^3J_{HH}$ = 8.1 Hz, 3H, H^1), 2.25 (s, 3H, H^5). MS (ESI$^+$, CH$_3$CN): m/z (%) = 262.13 (100, [tpe+H]$^+$), 545.24 (9, [2tpe+Na]$^+$).

Synthesis of [Cr(tpe)$_2$][BF$_4$]$_3$: Under oxygen-free conditions, tpe (500 mg, 1.91 mmol) was dissolved in a CH$_3$CN/H$_2$O mixture (50 ml, 1:1 v/v) and CrCl$_2$ (118 mg, 0.96 mmol) was added as a solid. The resulting dark green solution was stirred for 2 h at room temperature, heated to reflux for 3 h and stirred for 5 d at room temperature. [NH$_4$][BF$_4$] (312 mg, 2.98 mmol) dissolved in deaerated water (3 ml) was added to the reaction mixture. After stirring for 2 h under inert conditions, the mixture was left to stand for 16 h under air giving a pale-red solution. The solvents were removed under reduced pressure and the resulting red solid suspended in CH$_3$CN (5 ml). A colorless solid was removed by filtration. Diffusion of diethyl ether into the concentrated CH$_3$CN solution yielded golden crystals (495 mg, 0.59 mmol, 62 %). MS (ESI$^+$, CH$_3$CN): m/z (%) = 191.39 (18) [Cr(tpe)$_2$]$^{3+}$, 262.13 (12) [tpe+H]$^+$, 287.09 (60) [Cr(tpe)$_2$]$^{2+}$, 296.59 (13) [Cr(tpe)$_2$+F]$^{2+}$, 330.59 (100) [Cr(tpe)$_2$+BF$_4$]$^{2+}$, 364.05 (10), 576.15 (12), 593.19 (25)

$[Cr(tpe)_2+F]^+$, 639.20 (14), 661.19 (35) $[Cr(tpe)_2+(BF_4)]^+$, 706.19 (12), 748.20 (44) $[Cr(tpe)_2+(BF_4)_2]^+$. IR (ATR): $\tilde{\nu}$ = 3102 (w br, CH), 1601 (s), 1581 (w), 1492 (w), 1470 (s), 1441 (s), 1387 (w), 1307 (w), 1284 (w), 1294 (w), 1216 (w), 1175 (w), 1030 (vs br, BF), 847 (m), 773 (m), 758 (s), 750 (s), 721 (w), 660 (m), 639 (s), 564 (m), 519 (s), 449 (s), 400 (s) cm^{-1}. UV/Vis (CH_3CN): $\lambda(\varepsilon)$ = 431 (30), 329 (910), 274 (13700), 238 (30300 M^{-1} cm^{-1}) nm. Emission (CH_3CN; λ_{exc} = 436 nm): λ = 790, 749, 743, 727, 719 nm (structured band). Magnetism: χT = 1.875 cm^3 K mol^{-1} (6 – 300 K; solvate-free sample). CV (CH_3CN, $[N^nBu_4][BF_4]$, vs. FcH/FcH^+) = –0.88 (rev.), –1.54 (rev.), –2.49 (qrev.) V. Elemental analysis calcd. (%) for $C_{34}H_{30}B_3CrF_{12}N_6$ (835.06): C 48.90 H 3.62, N 10.06; found C 48.89, H 3.63 N 10.21.

Synthesis of $[Cr(tpe)_2][PF_6]_3$: Under oxygen-free conditions, tpe (99.4 mg, 0.38 mmol) was dissolved in H_2O (17 ml) and $CrCl_2$ (23.4 mg, 0.19 mmol) was added as a solid. The resulting dark green solution was stirred for 3 d at room temperature. $K[PF_6]$ (105 mg, 0.57 mmol) was dissolved in deaerated water (3 ml) and added to the reaction mixture. The solvent was removed under reduced pressure and the resulting solid dissolved in CH_3CN (3 ml). Diffusion of diethyl ether into the concentrated CH_3CN solution yielded dark green crystals (23 mg, 0.022 mmol, 12 %). MS (ESI^+, CH_3CN): *m/z* (%) = 144.98 (11), 287.09 (100) $[Cr(tpe)_2]^{2+}$, 359.57 (27) $[Cr(tpe)_2+PF_6]^{2+}$, 393.29 (10), 413.26 (11), 419.31 (10), 441.29 (27), 568.73 (6), 719.15 (69) $[Cr(tpe)_2+PF_6]^+$, 864.17 (6) $[Cr(tpe)_2+(PF_6)_2]^+$. IR (ATR): $\tilde{\nu}$ = 3102 (w br, CH), 2253 (w, CN), 1604 (m), 1470 (m), 1439 (m), 1389 (w), 1311 (w), 1218 (w), 1173 (w), 1113 (w), 1060 (m), 1037 (m), 824 (vs, PF), 775 (s), 762 (m), 660 (m), 639 (s), 555 (s, PF_2), 511 (m), 449 (m), 400 (m) cm^{-1}. Elemental analysis calcd. (%) for $C_{34}H_{30}CrF_{18}N_6P_3$ (1009.54)×0.5H_2O: C 40.09 H 3.07, N 8.25; found C 39.70, H 3.03 N 8.11.

Both salts crystallize with three acetonitrile solvate molecules according to single crystal XRD analyses (see below). However, the actual amount of solvent in a given sample depends on the grinding and drying procedure after crystallization.

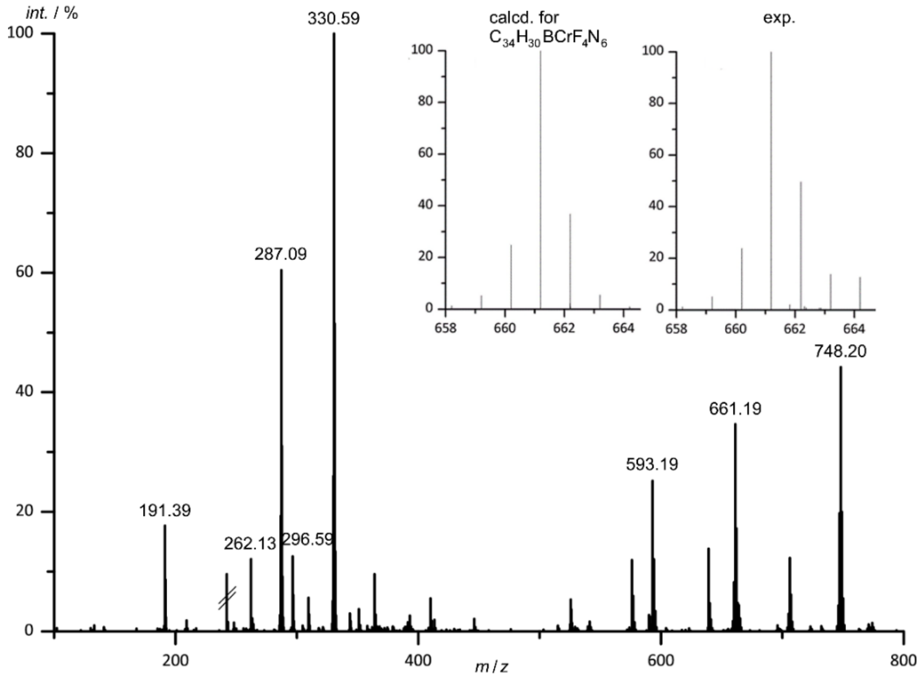

Figure S1. a) ESI$^+$ mass spectrum of $[Cr(tpe)_2][BF_4]_3$ in CH_3CN. The inset shows the calculated and experimental isotope distribution of the ion cluster $\{[Cr(tpe)_2][BF_4]\}^+$.

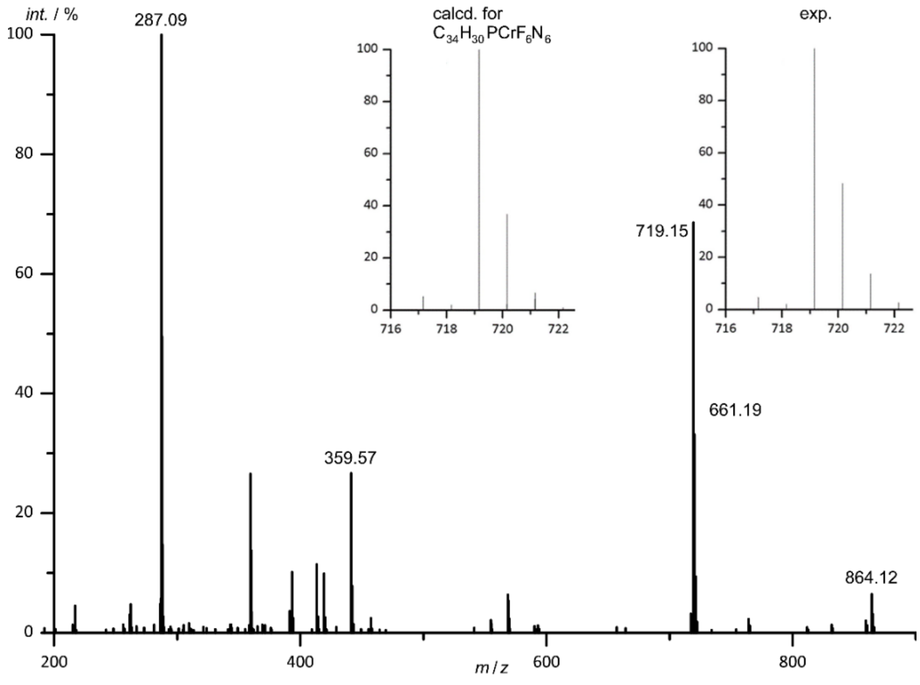

Figure S2. a) ESI$^+$ mass spectrum of $[Cr(tpe)_2][PF_6]_3$ in CH_3CN. The inset shows the calculated and experimental isotope distribution of the ion cluster $\{[Cr(tpe)_2][PF_6]\}^+$.

The cations of the salts are observed in the ESI$^+$ mass spectra as the trication $[Cr(tpe)_2]^{3+}$ and the dication $[Cr(tpe)_2]^{2+}$ and as ion clusters with the respective counter ions such as $\{[Cr(tpe)_2][X]\}^{2+}$ and $\{[Cr(tpe)_2][X]_2\}^+$ with $X^- = [BF_4]^-$, $[PF_6]^-$ or F^-.

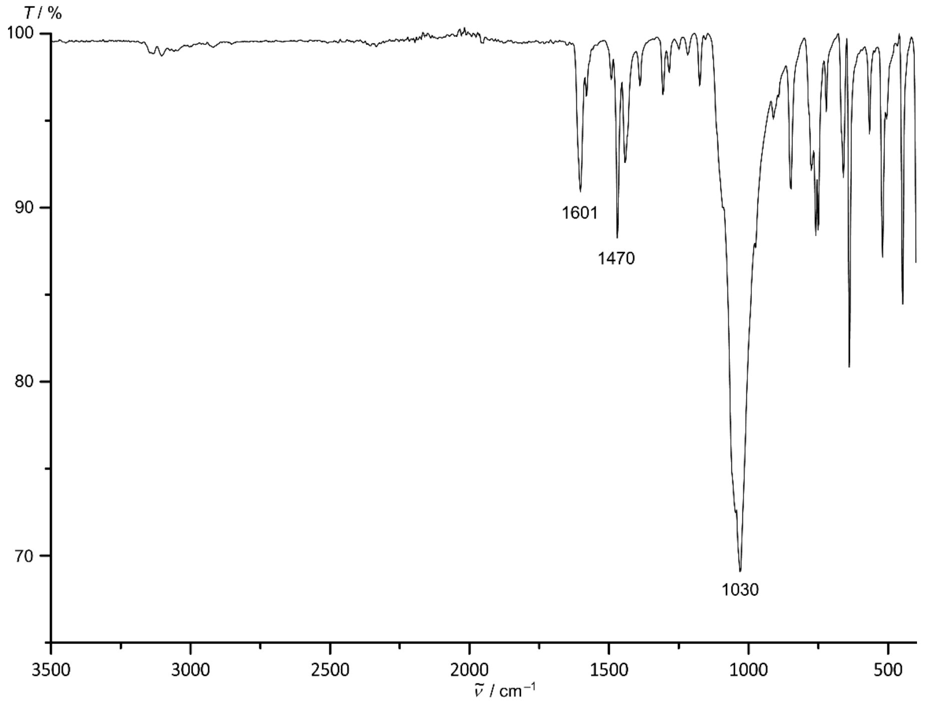

Figure S3. ATR-IR spectrum of $[Cr(tpe)_2][BF_4]_3$ (solvent-free sample).

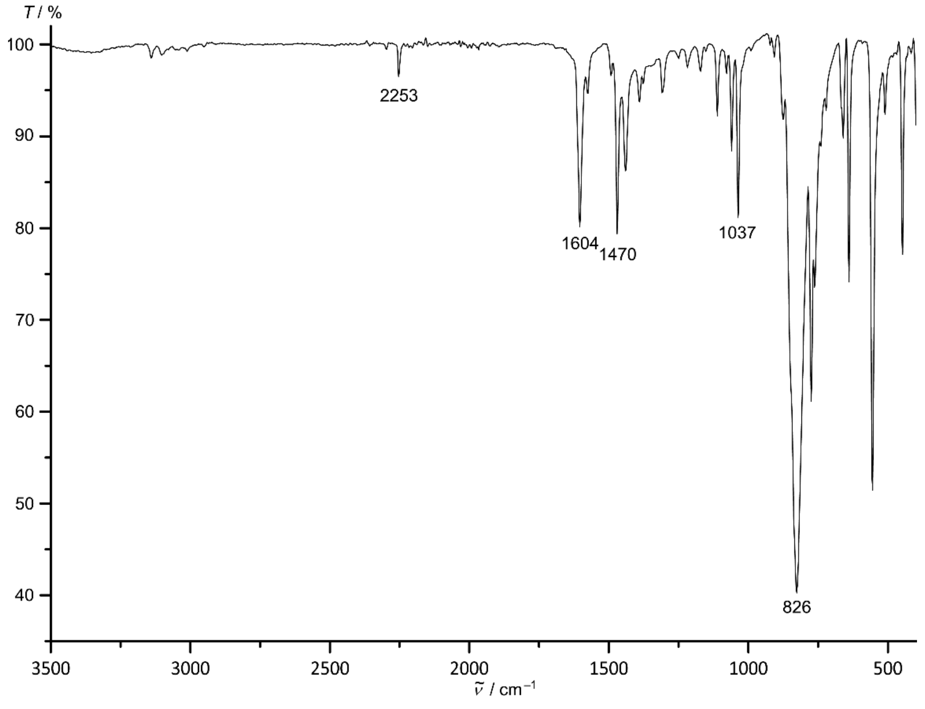

Figure S4. ATR-IR spectrum of $[Cr(tpe)_2][PF_6]_3 \times xCH_3CN$.

The counter ions show characteristic absorption bands for BF and PF stretching vibrations in the respective ATR-IR spectra. Crystals of *fac*-$[Cr(tpe)_2][PF_6]_3 \times 3CH_3CN$ display a sharp absorption band at 2253 cm^{-1} which is assigned to the CN stretching vibration of co-crystallized CH_3CN. The fingerprint region of both complex salts displays comparably few bands for CC and skeletal vibrations. This points to the high symmetry of the complex cations.

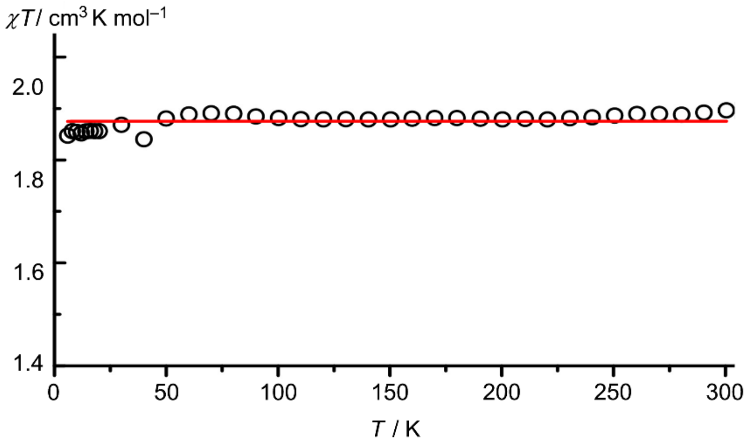

Figure S5. a) χT vs. T plot of $[Cr(tpe)_2][BF_4]_3$ (solvent-free sample); fit with $g = 2.000$ shown in red.

The temperature dependence of the magnetic susceptibility of a solvent-free sample of $[Cr(tpe)_2][BF_4]_3$ has been studied in the range 6 – 300 K in an external field of 0.1 T. In this temperature range, χT is essentially temperature independent and with 1.875 cm^3 K mol^{-1} very close to the spin-only value of a $S = {}^3/_2$ system ($^4A_{2g}$ ground state; $g = 2.000$; $\mu = 3.87\ \mu_B$). The fitted g value matches those of $[Cr(ddpd)_2][BF_4]_3$ and of $[Cr(tpy)_2][ClO_4]_3$.[S25-S27]

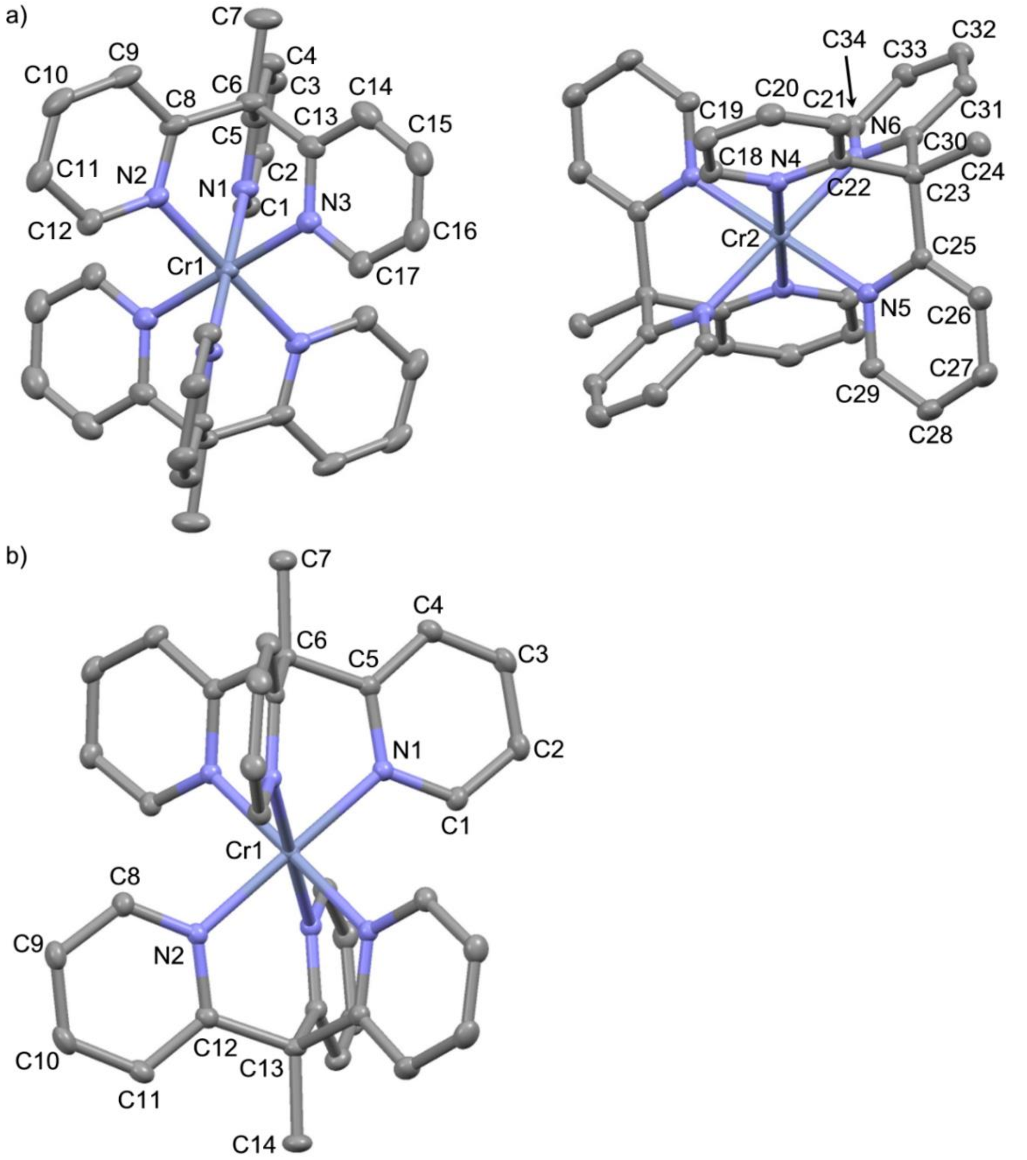

Figure S6. Plots of the cations of a) $[Cr(tpe)_2][BF_4]_3{\times}3CH_3CN$ with two independent cations and b) $[Cr(tpe)_2][PF_6]_3{\times}3CH_3CN$ with thermal ellipsoids set 30 % probability.

Table S1. Selected distances (Å) and angles (°) for $[Cr(tpe)_2][BF_4]_3 \times 3CH_3CN$,[a] $[Cr(tpe)_2][PF_6]_3 \times 3CH_3CN$,[b] and $[Cr(tpe)_2]^{3+}$ (DFT).

	$[Cr(tpe)_2][BF_4]_3 \times 3CH_3CN$	$[Cr(tpe)_2][PF_6]_3 \times 3CH_3CN$	$[Cr(tpe)_2]^{3+}$
Cr1-N1	2.041(2)	2.046(3) / 2.044(3)	2.055 / 2.055
Cr1-N2	2.041(2)	2.046(3) / 2.044(3)	2.055 / 2.055
Cr1-N3	2.031(2)	2.046(3) / 2.044(3)	2.053 / 2.053
tpe (intraligand)			
N1-Cr1-N2	86.34(8)	86.48(11) / 86.08(11)	86.74 / 86.78
N1-Cr1-N3	86.66(9)	86.48(11) / 86.08(11)	86.73 / 86.73
N2-Cr1-N3	86.57(9)	86.48(11) / 86.08(11)	86.79 / 86.75
tpe (interligand)			
N1-Cr1-N1’	180.0	93.22(10)	179.96
N1-Cr1-N2’	93.66(8)	179.22(12)	93.26
N1-Cr1-N3’	93.34(9)	94.22(10)	93.29
N2-Cr1-N2’	180.0	94.22(10)	179.99
N2-Cr1-N3’	93.43(9)	93.22(10)	93.27
N3-Cr1-N3’	180.0	179.22(12)	179.95
Cr2-N4	2.052(2)	–	–
Cr2-N5	2.048(2)	–	–
Cr2-N6	2.049(2)	–	–
N4-Cr2-N5	86.00(8)	–	–
N4-Cr2-N6	86.85(8)	–	–
N5-Cr2-N6	86.29(8)	–	–
N4-Cr2-N4’	180.0	–	–
N4-Cr2-N5’	93.99(8)	–	–
N4-Cr2-N6’	93.15(8)	–	–
N5-Cr2-N5’	180.0	–	–
N5-Cr2-N6’	93.71(8)	–	–
N6-Cr2-N6’	180.0	–	–
Cr1···N(solvent)	4.47 (2×)	4.53 (3×)	–
Cr1···F(counterion)	4.46 (2×), 4.55 (2×)	4.64 (3×)	–
Cr2···N(solvent)	4.45 (2×), 5.20 (2×)	–	–
Cr2···F(counterion)	4.42 (2×)	–	–

[a] Two independent centrosymmetric trications Cr1/Cr2 in the unit cell.

[b] A single trication in the unit cell with two crystallographically independent C_3 symmetric tpe ligands.

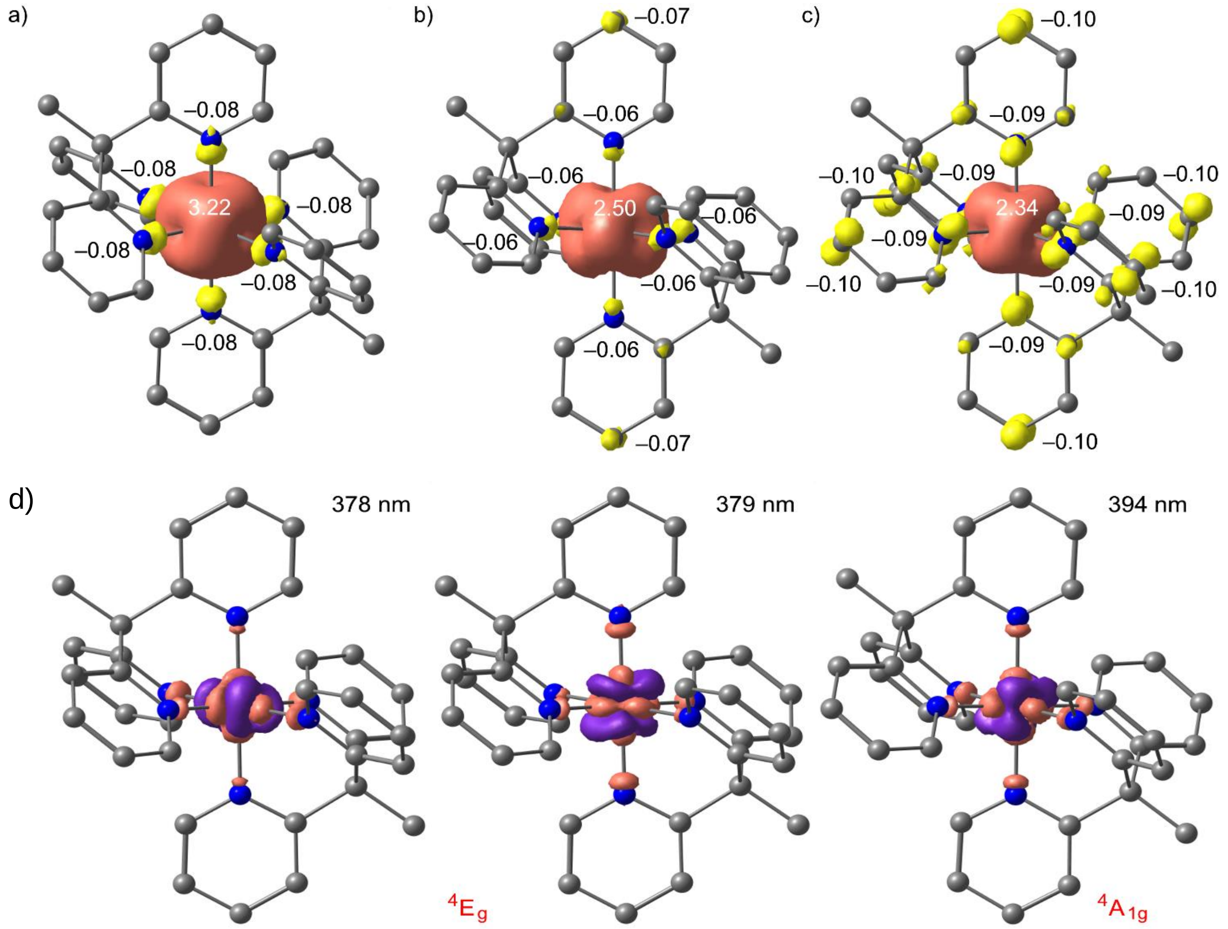

Figure S7. DFT optimized geometries of a) $[Cr(tpe)_2]^{3+}$ (quartet state), b) $[Cr(tpe)_2]^{2+}$ (triplet state) and c) $[Cr(tpe)_2]^{+}$ (doublet state), and corresponding Mulliken spin densities plotted at 0.006 a.u. with the α spin in orange and the β spin in yellow. d) Three lowest TD-DFT calculated spin-allowed transitions; these correspond to the spin-allowed $^4A_{2g}\rightarrow{}^4E_g$ and $^4A_{2g}\rightarrow{}^4A_{1g}$ ligand field transitions (in D_{3d} symmetry). Hydrogen atoms omitted. Difference electron densities plotted with an isosurface value of 0.01 a.u.; purple = depletion; orange = gain.

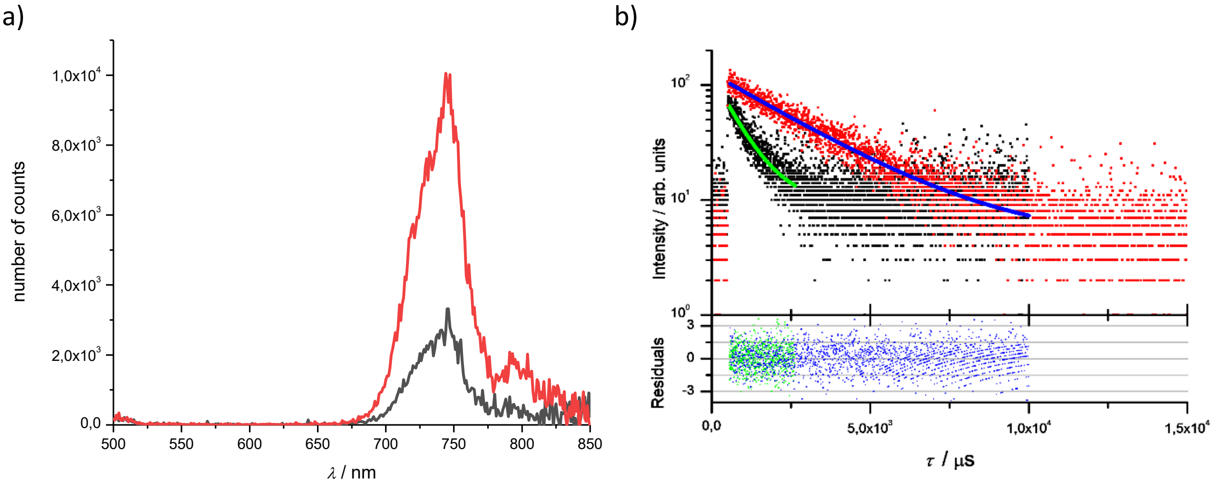

Figure S8. a) Emission spectra of $[Cr(tpe)_2][BF_4]_3$ in $H_2O/HClO_4$ at room temperature (λ_{exc} = 428 nm) under inert (red) and air-saturated conditions (black) and b) corresponding emission decay curves.

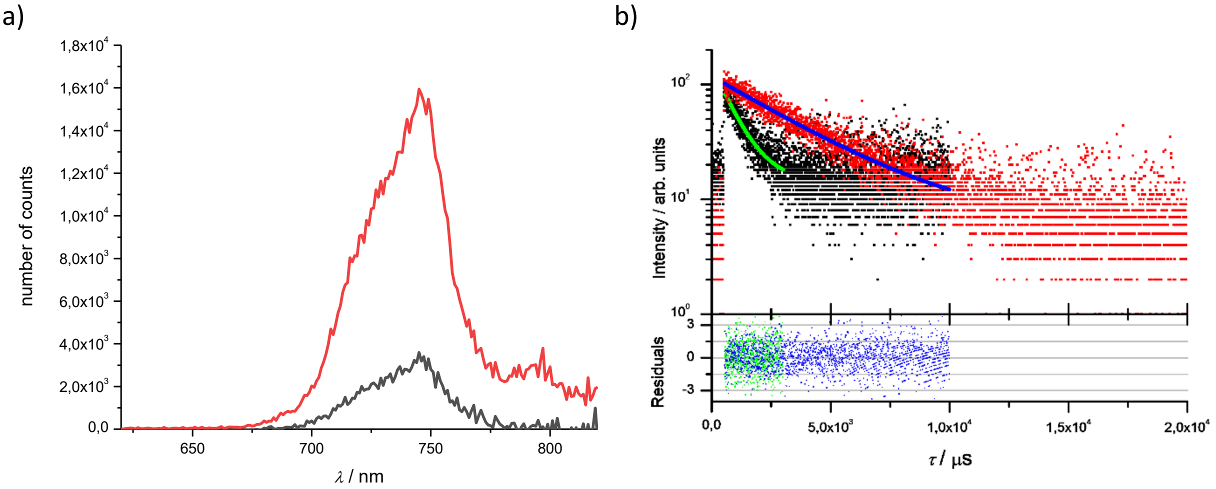

Figure S9. a) Emission spectra of $[Cr(tpe)_2][BF_4]_3$ in $D_2O/DClO_4$ at room temperature (λ_{exc} = 428 nm) under inert (red) and air-saturated conditions (black) and b) corresponding emission decay curves.

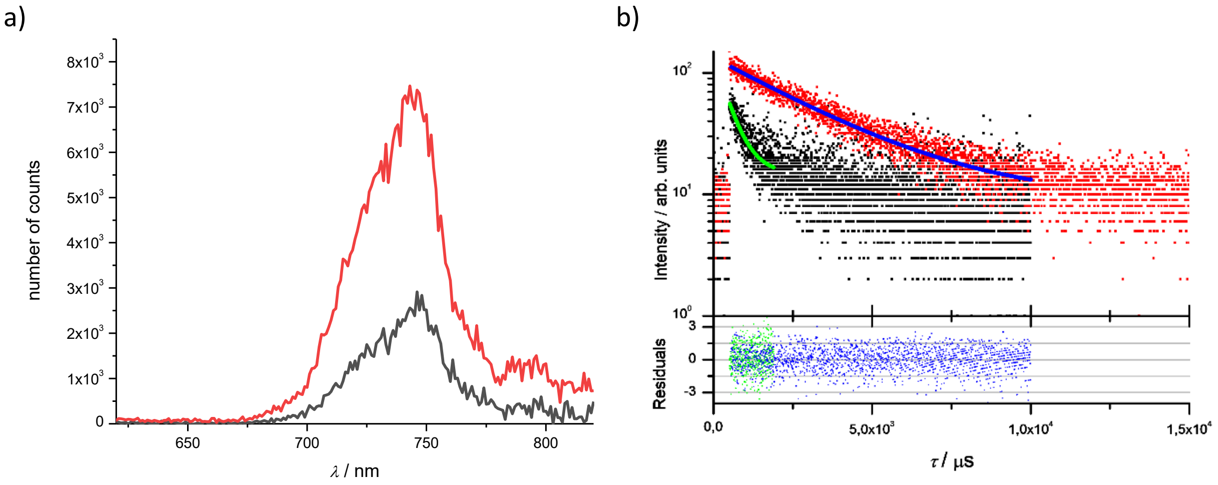

Figure S10. a) Emission spectra of $[Cr(tpe)_2][BF_4]_3$ in $CH_3CN/HClO_4$ at room temperature (λ_{exc} = 428 nm) under inert (red) and air-saturated conditions (black) and b) corresponding emission decay curves.

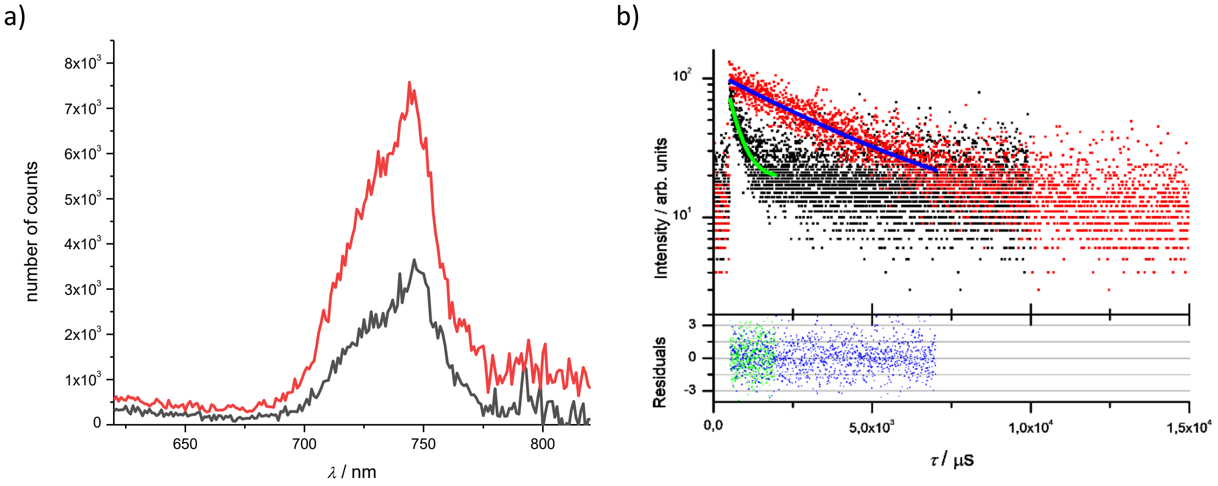

Figure S11. a) Emission spectra of $[Cr(tpe)_2][BF_4]_3$ in $CD_3CN/DClO_4$ at room temperature (λ_{exc} = 428 nm) under inert (red) and air-saturated conditions (black) and b) corresponding emission decay curves.

a) b)

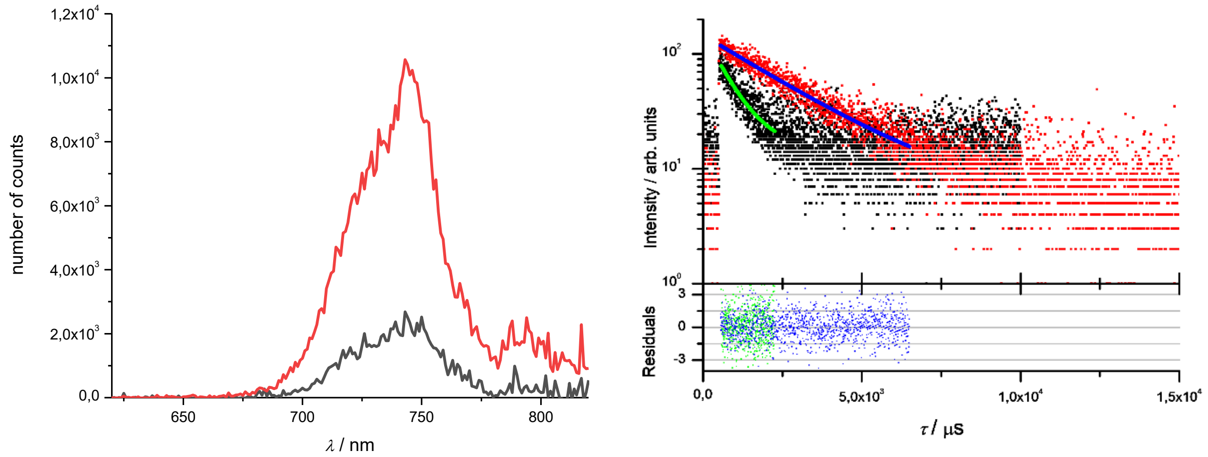

Figure S12. a) Emission spectra of $[Cr(tpe)_2][BF_4]_3$ in $H_2O/NaClO_4$ at room temperature (λ_{exc} = 428 nm) under inert (red) and air-saturated conditions (black) and b) corresponding emission decay curves.

a) b)

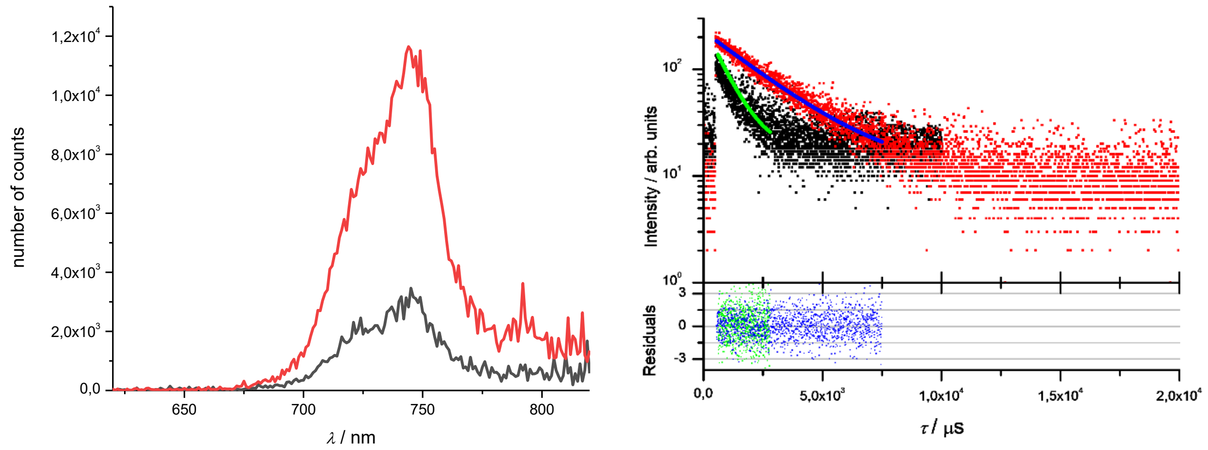

Figure S13. a) Emission spectra of $[Cr(tpe)_2][BF_4]_3$ in $D_2O/NaClO_4$ at room temperature (λ_{exc} = 428 nm) under inert (red) and air-saturated conditions (black) and b) corresponding emission decay curves.

Table S2. Quantum yields Φ, luminescence lifetimes τ and radiative lifetimes τ_r of $[Cr(tpe)_2][BF_4]_3$ and $[Cr(ddpd)_2][BF_4]_3$ [S29] under inert and under air-saturated conditions ($\tau_r = \tau / \Phi$). $[Cr(tpe)_2][BF_4]_3$ was measured in the presence of $HClO_4$, $DClO_4$ or $NaClO_4$ (0.1 M), respectively, while no acid was employed for $[Cr(ddpd)_2][BF_4]_3$. Please note, that the solubility of O_2 in H_2O changes with pH so that the quenching data of $[Cr(tpe)_2][BF_4]_3$ and $[Cr(ddpd)_2][BF_4]_3$ should not be compared directly (Figures S8 – S13).

	$[Cr(tpe)_2][BF_4]_3$ (inert)	$[Cr(tpe)_2][BF_4]_3$ (O_2-sat.)	$[Cr(ddpd)_2][BF_4]_3$ (inert)	$[Cr(ddpd)_2][BF_4]_3$ (O_2-sat.)
Φ($H_2O/HClO_4$) / %	5.4	2.2	11.0	2.1
τ($H_2O/HClO_4$) / µs	2800	780	898	177
τ_r($H_2O/HClO_4$) / ms	51.9	35.5	8.2	8.4
Φ($D_2O/DClO_4$) / %	8.2	4.1	14.2	2.0
τ($D_2O/DClO_4$) / µs	4500	890	1164	150
τ_r($D_2O/DClO_4$) / ms	54.9	21.7	8.2	7.5
Φ($CH_3CN/HClO_4$) / %	6.1	3.4	12.1	0.60
τ($CH_3CN/HClO_4$) / µs	2900	320	899	44
τ_r($CH_3CN/HClO_4$) / ms	47.5	9.4	7.4	7.3
Φ($CD_3CN/DClO_4$) / %	7.7	4.5	11.7	0.60
τ($CD_3CN/DClO_4$) / µs	3300	360	810	24
τ_r($CD_3CN/DClO_4$) / ms	42.9	8.0	6.9	4.0
Φ($H_2O/NaClO_4$) / %	4.2	2.1		
τ($H_2O/NaClO_4$) / µs	2500	760		
τ_r($H_2O/NaClO_4$) / ms	59.5	36.2		
Φ($D_2O/NaClO_4$) / %	6.3	4.1		
τ($D_2O/NaClO_4$) / µs	3900	880		
τ_r($D_2O/NaClO_4$) / ms	61.9	21.5		

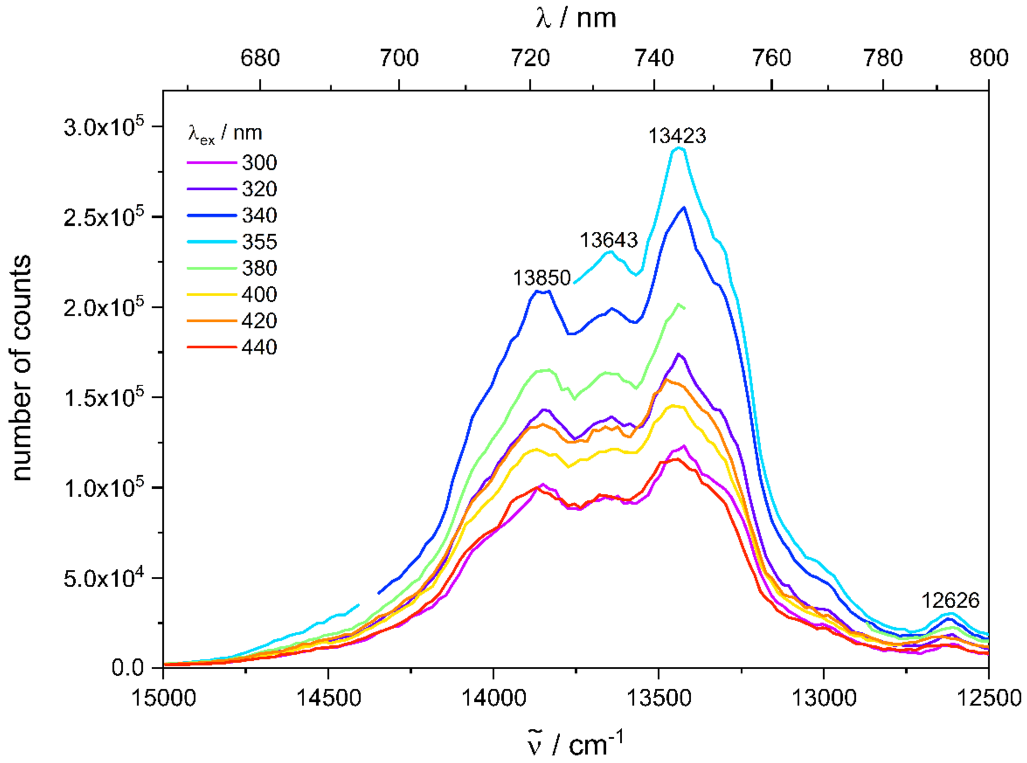

Figure S14. Emission spectra of $[Cr(tpe)_2][BF_4]_3$ as KBr disk at 290 K with different excitation wavelengths.

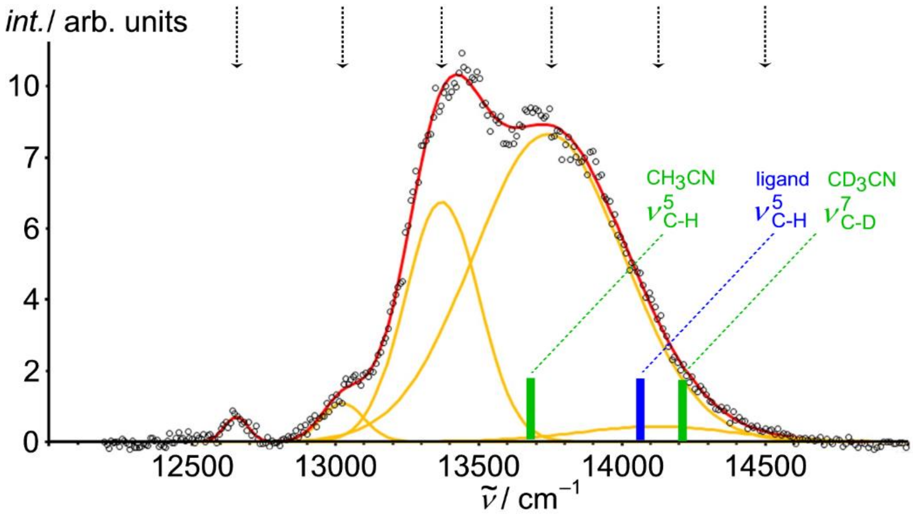

Figure S15. Room temperature emission band of $[Cr(tpe)_2][BF_4]_3$ in CH_3CN (λ_{exc} = 436 nm) and a fit of the band envelope by five Gaussians (12653, 13026, 13372, 13744, 14120 cm^{-1}; R^2 = 0.99684). The energies of vibrational C-H(ligand), C-H(CH_3CN) and C-D(CD_3CN) overtones relevant for multiphonon relaxation are indicated by blue and green bars.

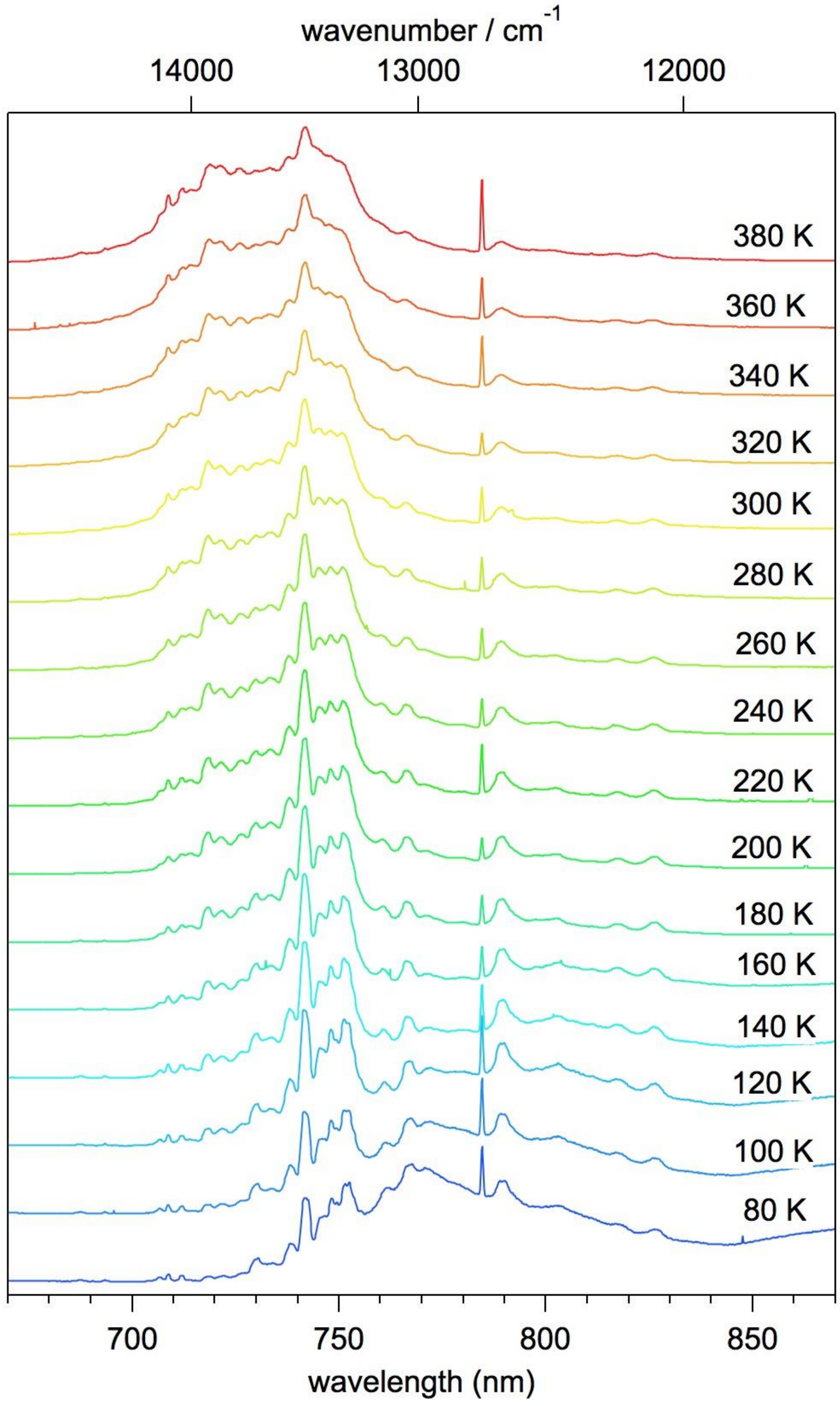

Figure S16. Emission spectra of crystals of $[Cr(tpe)_2][BF_4]_3$ at different temperatures (λ_{exc} = 488 nm).

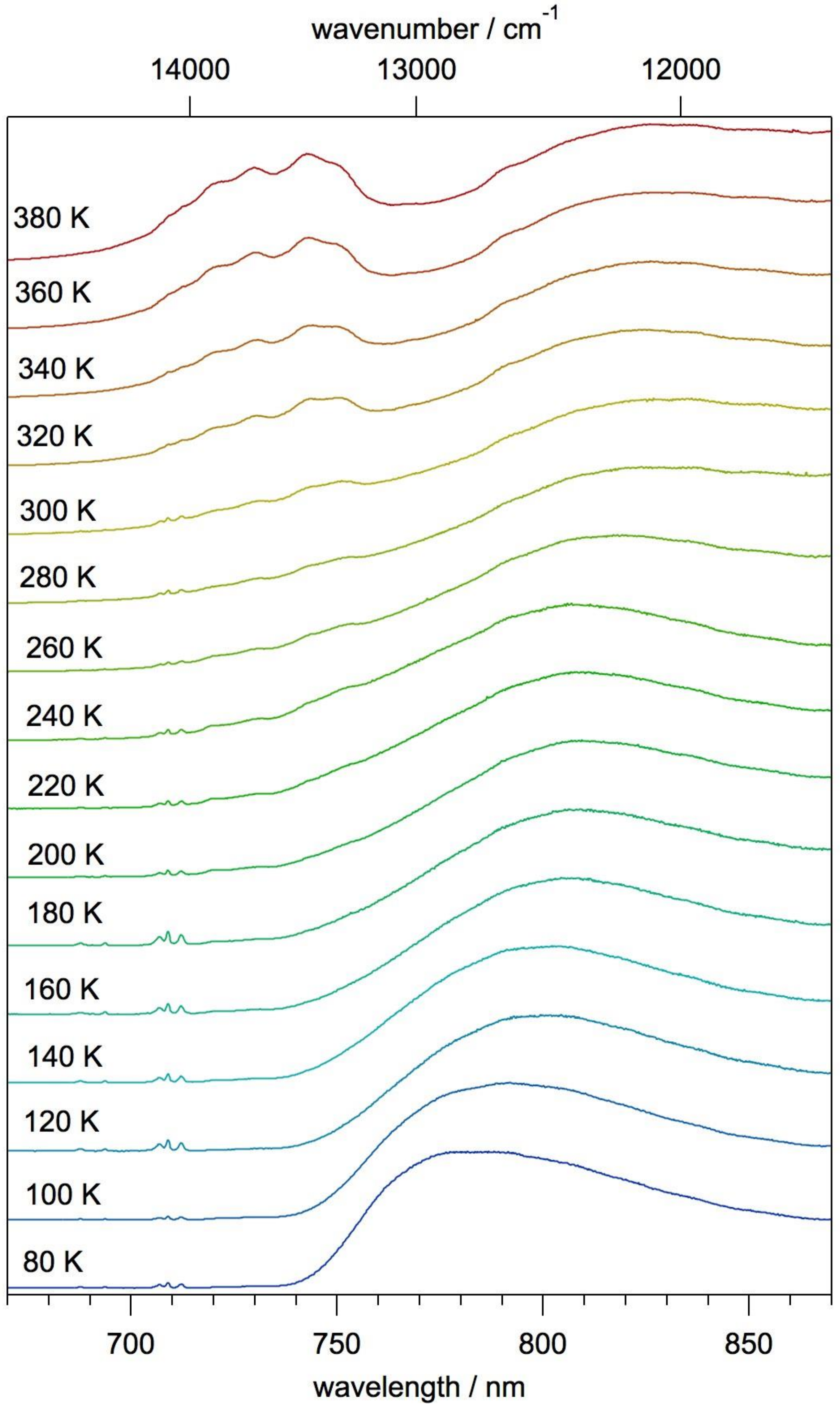

Figure S17. Emission spectra of crystals of $[Cr(tpe)_2][PF_6]_3$ at different temperatures (λ_{exc} = 488 nm).

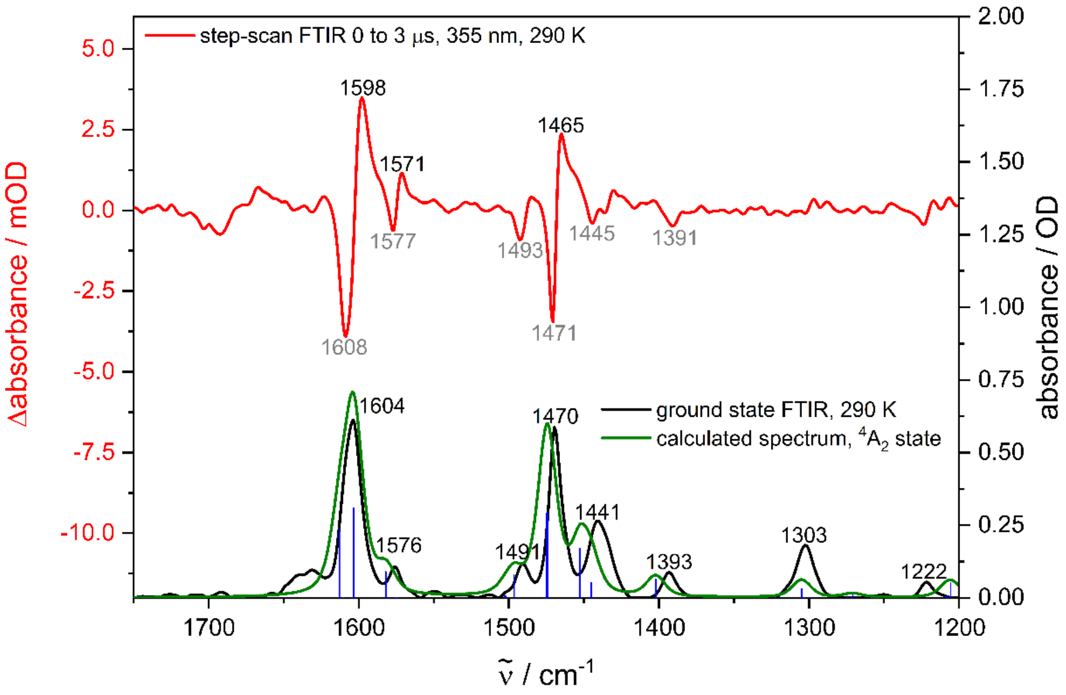

Figure S18. Step-scan FT-IR spectrum (red) and ground state IR spectrum (black) of $[Cr(tpe)_2][BF_4]_3$ as KBr disk at 290 K and DFT calculated IR absorption bands (blue, scaled by 0.98) and DFT calculated IR spectrum (green, pseudo-Voigt functions with FWHM = 15 cm^{-1}).

Table S3. Excited state lifetimes of $[Cr(tpe)_2][BF_4]_3$ as obtained from KBr disk or from a neat film. For the measurements at 290 K both the results of the tri- and tetraexponential fits are given.

method	medium	*T* / K	τ_1 / ns (fraction / %)	τ_2 / ns (fraction / %)	τ_3 / µs (fraction / %)	τ_4 / µs (fraction / %)
step-scan	KBr	290	-	-	0.71 ± 0.07 (35)	10 ± 2 (65)
step-scan	KBr	20	-	-	2.2 ± 0.2 (18)	66 ± 7 (82)
TCSPC λ_{exc} = 389 nm λ_{em} = 745 nm	KBr	290	-	88 ± 2 (8)	0.71 ± 0.01 (21)	10.9 ± 0.1 (71)
TCSPC λ_{exc} = 389 nm λ_{em} = 745 nm	KBr	290	38 ± 3 (2)	276 ± 8 (10)	1.49 ± 0.05 (17)	14.4 ± 0.3 (71)
TCSPC λ_{exc} = 389 nm λ_{em} = 733 nm	KBr	290	-	96 ± 1 (8)	0.72 ± 0.01 (22)	9.5 ± 0.1 (70)
TCSPC λ_{exc} = 389 nm λ_{em} = 733 nm	KBr	290	47 ± 2 (3)	305 ± 8 (12)	1.57 ± 0.05 (19)	13.7 ± 0.3 (67)
TCSPC λ_{exc} = 389 nm λ_{em} = 722 nm	KBr	290	-	105 ± 2 (8)	0.83 ± 0.02 (21)	12.2 ± 0.3 (72)
TCSPC λ_{exc} = 389 nm λ_{em} = 722 nm	KBr	290	58 ± 4 (3)	310 ± 20 (9)	1.5 ± 0.1 (17)	15.3 ± 0.5 (70)
TCSPC λ_{exc} = 389 nm λ_{em} = 759 nm	KBr	20	-	-	0.5 ± 0.1 (2)	55 ± 13 (98)
TCSPC λ_{exc} = 389 nm λ_{em} = 740 nm	KBr	20	-	-	0.60 ± 0.07 (5)	50 ± 10 (95)
TCSPC λ_{exc} = 389 nm λ_{em} = 740 nm	Neat film	290	-	190 ± 5 (8)	1.30 ± 0.05 (21)	12.0 ± 0.4 (71)
TCSPC λ_{exc} = 389 nm λ_{em} = 740 nm	Neat film	290	47 ± 7 (1)	290 ± 20 (9)	1.5 ± 0.1 (19)	13.5 ± 0.5 (72)
TCSPC λ_{exc} = 389 nm λ_{em} = 717 nm	Neat film	290		172 ± 6 (5)	0.94 ± 0.03 (21)	10.6 ± 0.2 (74)
TCSPC λ_{exc} = 389 nm λ_{em} = 717 nm	Neat film	290	120 ± 20 (2)	400 ± 50 (10)	1.7 ± 0.1 (19)	12.5 ± 0.2 (69)

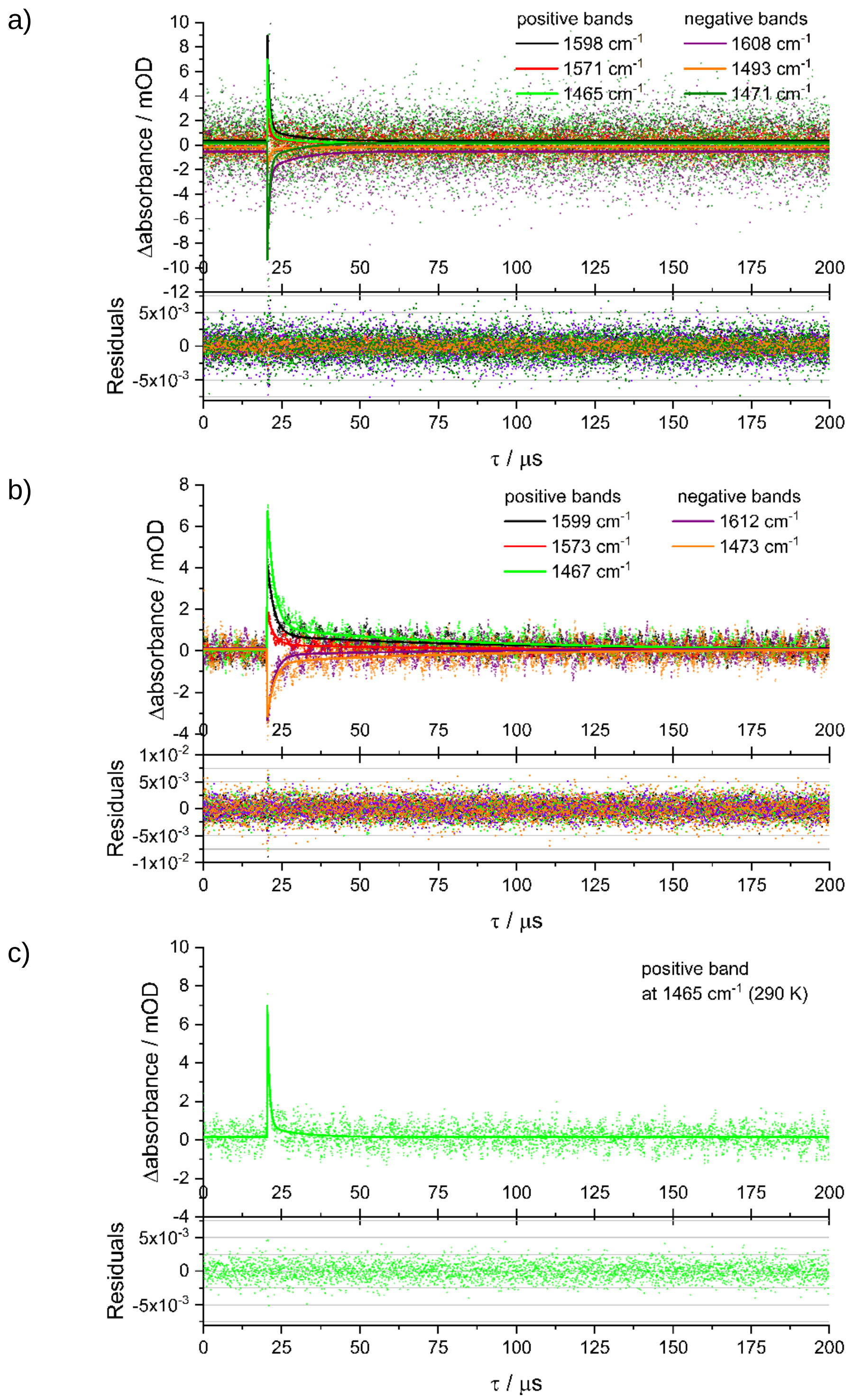
a)
positive bands
1598 cm-1
1571 cm-1
1465 cm-1
negative bands
1608 cm-1
1493 cm-1
1471 cm-1
Δabsorbance / mOD
Residuals
τ / µs
b)
positive bands
1599 cm-1
1573 cm-1
1467 cm-1
negative bands
1612 cm-1
1473 cm-1
Δabsorbance / mOD
Residuals
τ / µs
c)
positive band
at 1465 cm-1 (290 K)
Δabsorbance / mOD
Residuals
τ / µs

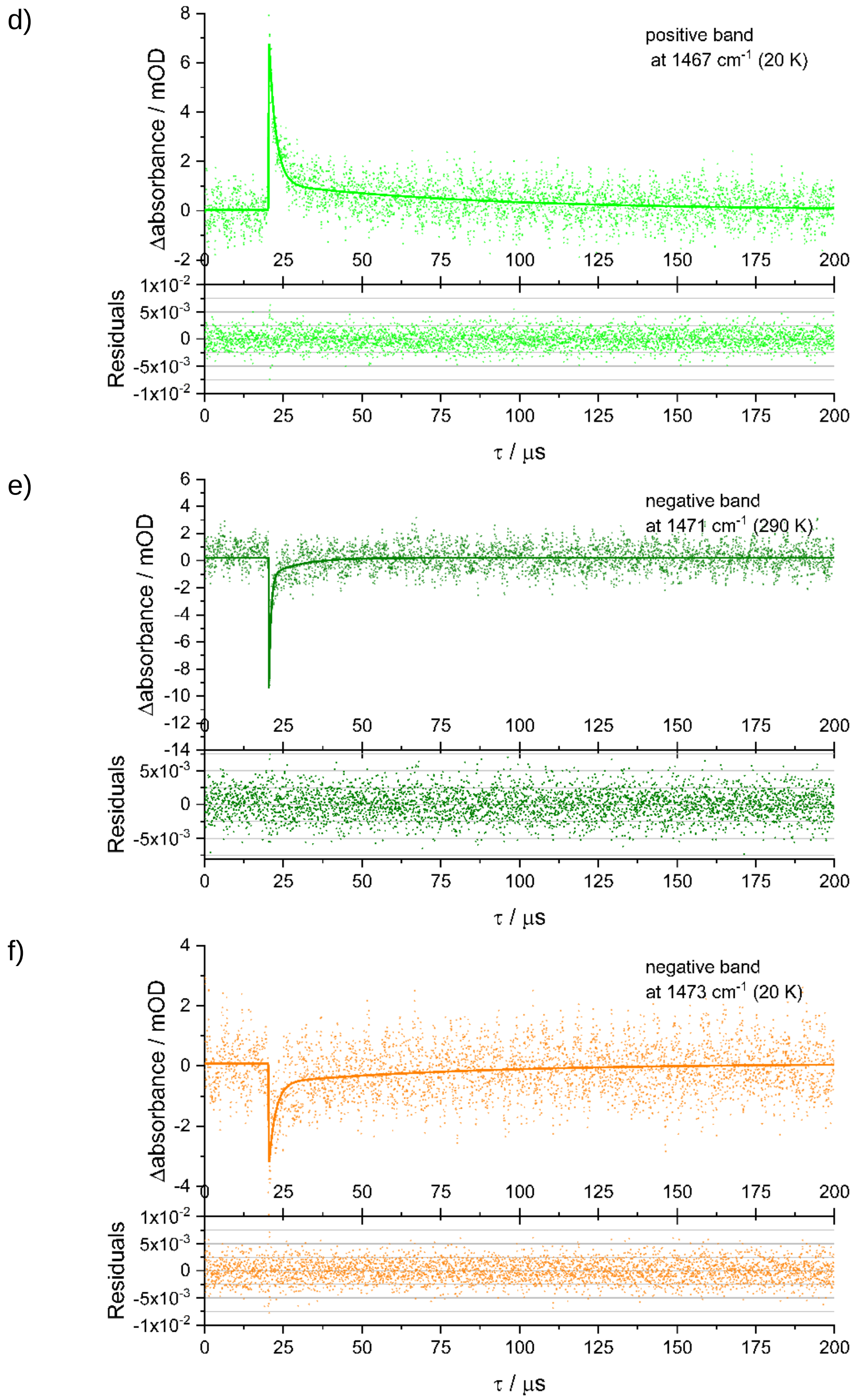

Figure S19. Decay curves and global fits obtained from step-scan FT-IR data at a) 290 K and b) 20 K including residuals. c) – f) Fits of the transition around 1465 cm^{-1} at 290 K and 20 K (positive band that belongs to the excited state) and transition around 1470 cm^{-1} at 290 K and 20 K (negative band that belongs to the electronic ground state) including residuals.

Figure S20. Decay curves as well as a) tri- and b) tetraexponential fits obtained from TCSPC data at 290 K (λ_{obs} = 722 nm, KBr). The offset results from the dark current.

a)

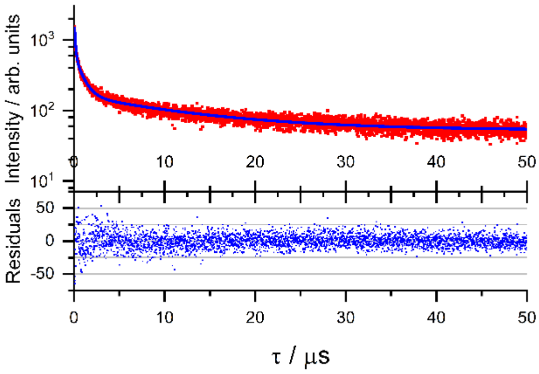

b)

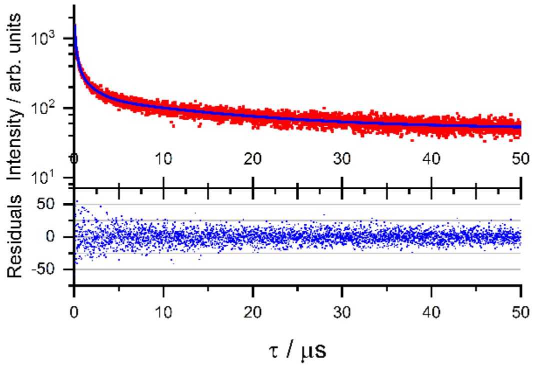

Figure S21. Decay curves as well as a) tri- and b) tetraexponential fits obtained from TCSPC data at 290 K (λ_{obs} = 733 nm, KBr). The offset results from the dark current.

a)

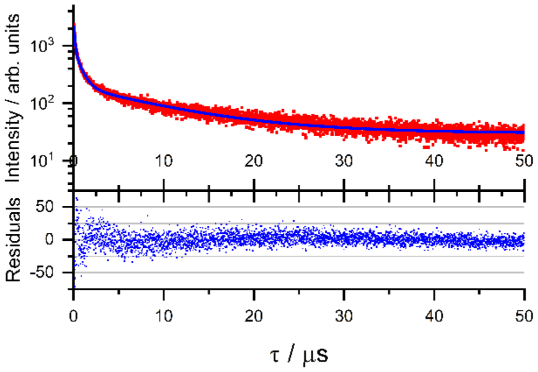

b)

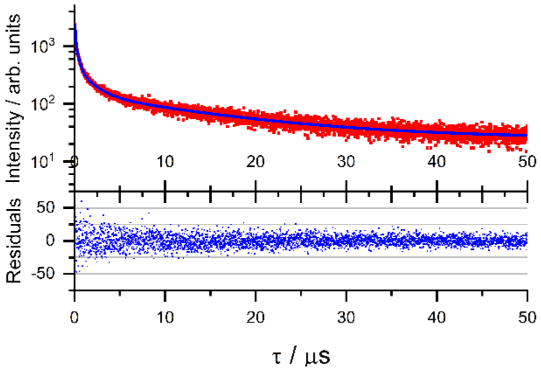

Figure S22. Decay curves as well as a) tri- and b) tetraexponential fits obtained from TCSPC data at 290 K (λ_{obs} = 745 nm, KBr). The offset results from the dark current.

a)

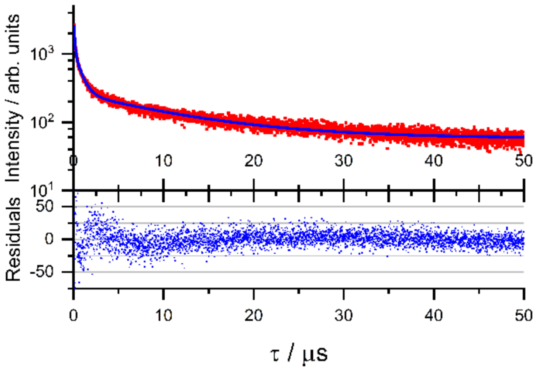

b)

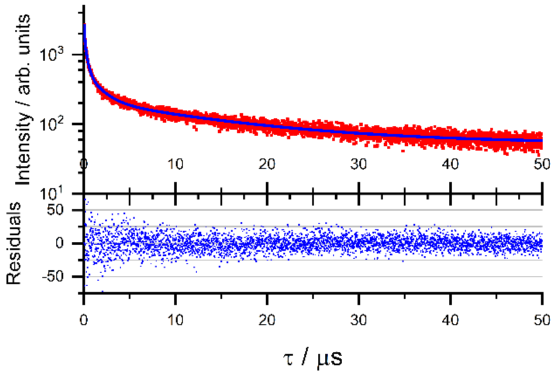

a)

b)

c)

d)

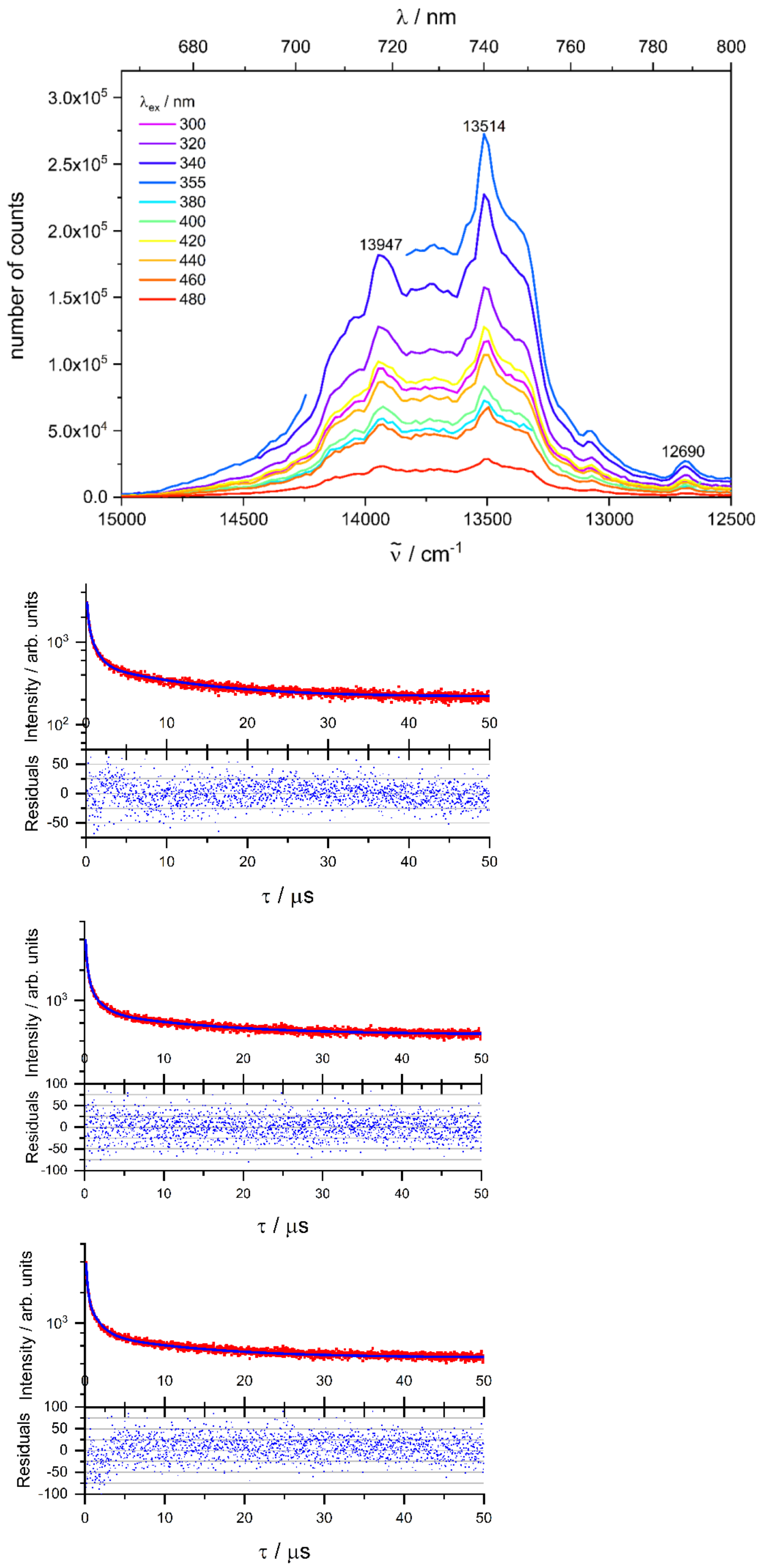

λ / nm
680
700
720
740
760
780
800
λ_{ex} / nm
300
320
340
355
380
400
420
440
460
480
13514
13947
12690
number of counts
3.0x10^5
2.5x10^5
2.0x10^5
1.5x10^5
1.0x10^5
5.0x10^4
0.0
15000
14500
14000
13500
13000
12500
ṽ / cm-1
Intensity / arb. units
Residuals
10^3
10^2
100
50
0
-50
-100
0
10
20
30
40
50
τ / μs

e)

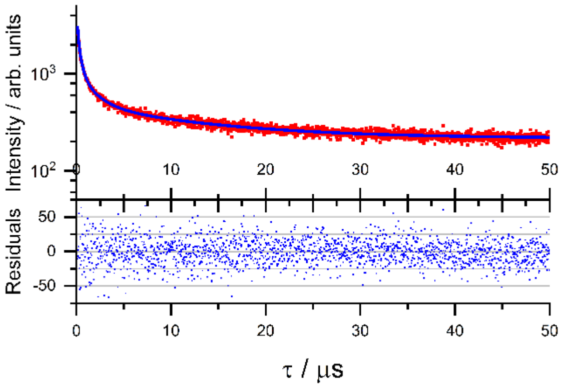

Figure S23. a) Emission spectra of $[Cr(tpe)_2][BF_4]_3$ as neat film at 290 K with different excitation wavelengths, decay curves as well as b) tri- and c) tetraexponential fits obtained from TCSPC data at 290 K (λ_{obs} = 717 nm), decay curves as well as d) tri- and e) tetraexponential fits obtained from TCSPC data at 290 K (λ_{obs} = 740 nm).

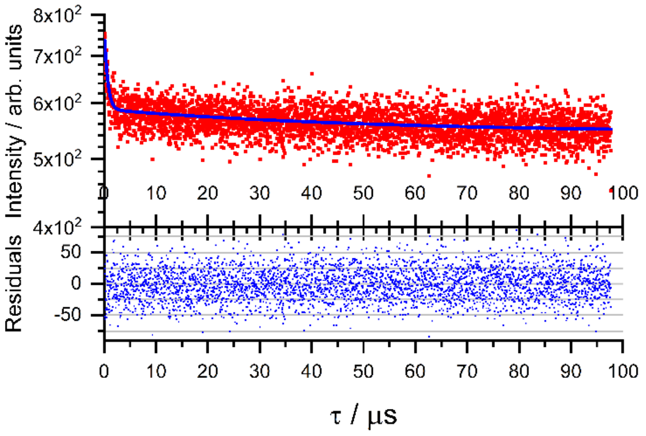

Figure S24. Decay curves and biexponential fits obtained from TCSPC data at 20 K (λ_{obs} = 740 nm, KBr).

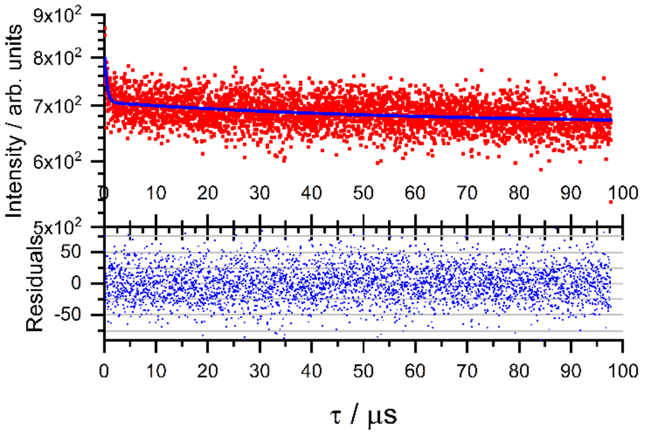

Figure S25. Decay curves and biexponential fits obtained from TCSPC data at 20 K (λ_{obs} = 759 nm, KBr).

Spectral overlap integral (SOI) calculations for $[Cr(tpe)_2]^{3+}$ / CH_3CN and CD_3CN:

The C-H overtone bands of CH_3CN in solution (Figures S26 and S27) were measured in the relevant spectral region (11500 to 16000 cm^{-1} ≡ fourth C-H stretch overtone and third combination region) corresponding to the spectral region of the doublet emission of $[Cr(tpe)_2]^{3+}$. The obtained data are consistent with previous measurements in liquid CH_3CN and show that the band intensities are largely dominated by C-H stretching overtones without significant participation of C≡N vibrations.[S28] The measured bands were fitted by a series of Gaussian functions in order to extract a coherent expression of the band shape for the SOI calculation (vide infra).

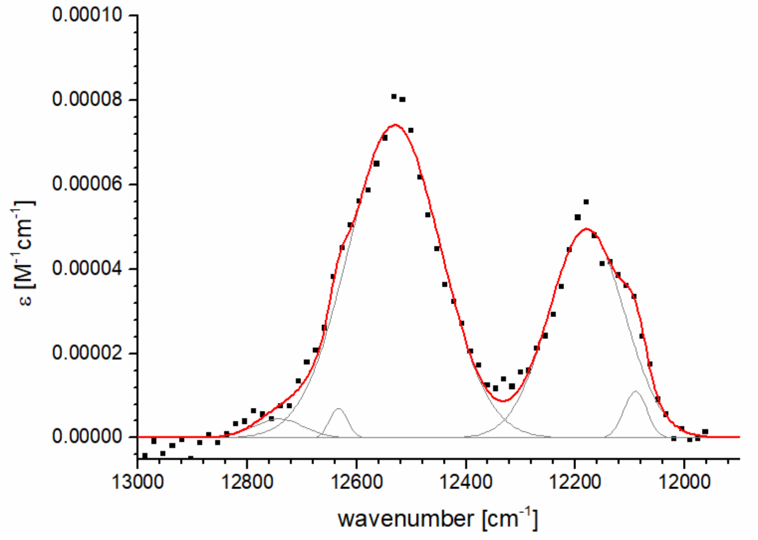

Figure S26. Near-IR absorption spectrum of neat CH_3CN (black, c = 19.15 M, d = 5 cm) in the third C-H combination region. Gaussian fit functions for the analytical extraction of the band shape (grey: individual Gaussians; red: sum of the Gaussians).

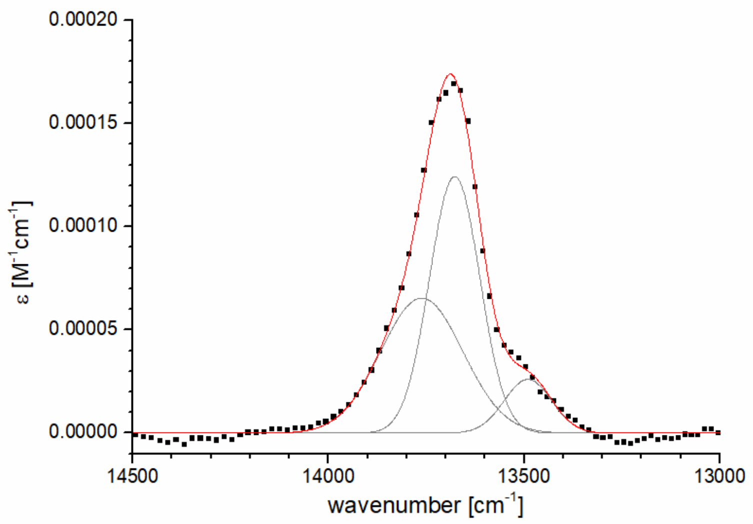

Figure S27. Near-IR absorption spectrum of neat CH_3CN (black, c = 19.15 M, d = 5 cm) in the fourth C-H overtone region (vibrational quantum number ν = 5). Gaussian fit functions for the analytical extraction of the band shape (grey: individual Gaussians; red: sum of the Gaussians).

The C-D absorption bands in the same region as for the C-H overtones (11500 to 16000 cm^{-1}) could not be reliably measured due to the extremely small molar extinction values associated with these overtones. In order to be able to calculate the corresponding SOI with the chromium emission, the spectral characteristics of the required higher overtones C-D (in particular the overtones ν = 6 and 7) were extrapolated from the systematic changes seen in the lower overtones (ν = 3 – 5) that could be measured. For this purpose, each measured overtone band was fitted with a single Gaussian function of the form $y(x) = A \cdot \exp(-0.5*((x-x_c)/\sigma)^2)$ defined by amplitude A, center wavenumber x_c, and Gaussian width σ. As can be seen from Figures S28–S30, this is a rather crude approximation for the lower overtone bands (especially seen in Figure S28) but improves with increasing vibrational quantum number as the vibrations gain more local-mode character and coupling between oscillators is diminishing.

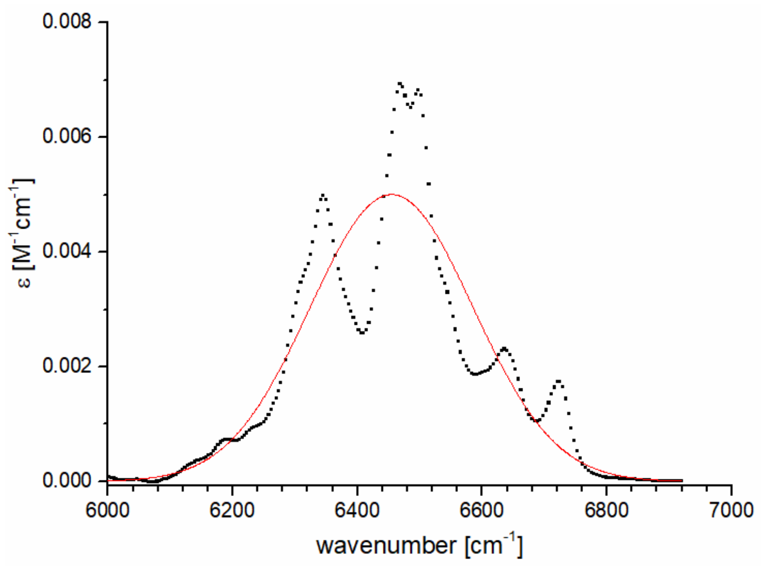

Figure S28. Near-IR absorption spectrum of neat CD_3CN (black, *c* = 19.15 M, *d* = 5 cm) in the third C-D combination region. Single Gaussian fit function (red) for the extrapolation procedure to higher overtones (see text).

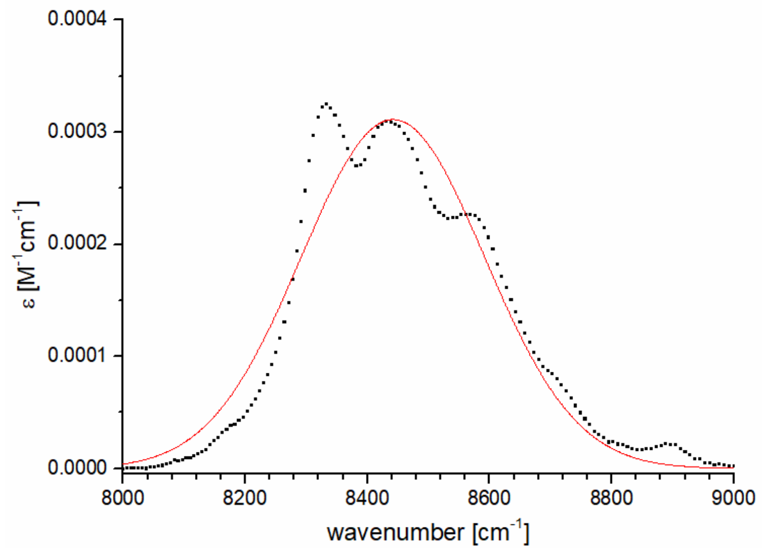

Figure S29. Near-IR absorption spectrum of neat CD_3CN (black, *c* = 19.15 M, *d* = 5 cm) in the fourth C-D combination region. Single Gaussian fit function (red) for the extrapolation procedure to higher overtones (see text).

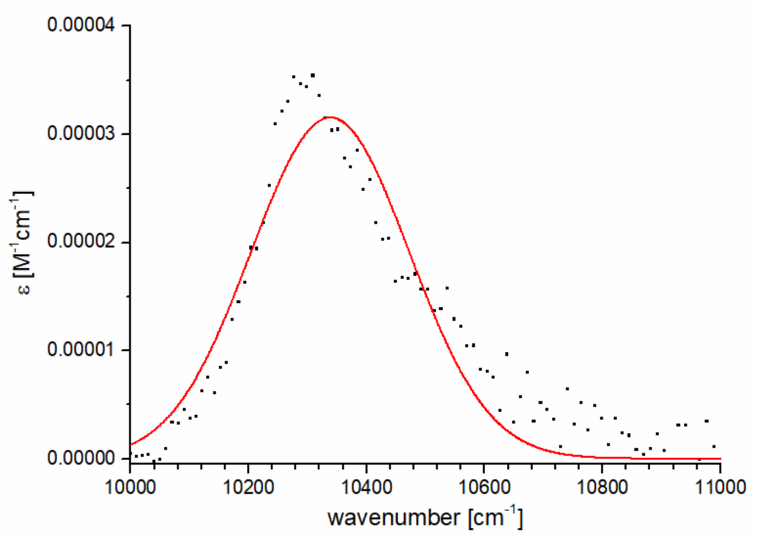

Figure S30. Near-IR absorption spectrum of neat CD_3CN (black, c = 19.15 M, d = 5 cm) in the fifth C-D combination region. Single Gaussian fit function (red) for the extrapolation procedure to higher overtones (see text).

Due to the high degree of coupling in the overtones, the only Gaussian parameter that turned out to be reliable for the extrapolation was the amplitude A. As already successfully shown in previous work,[S29] the semilogarithmic plot of log(A) vs. vibrational quantum number ν could be fitted well by a linear relationship (Figure S31).

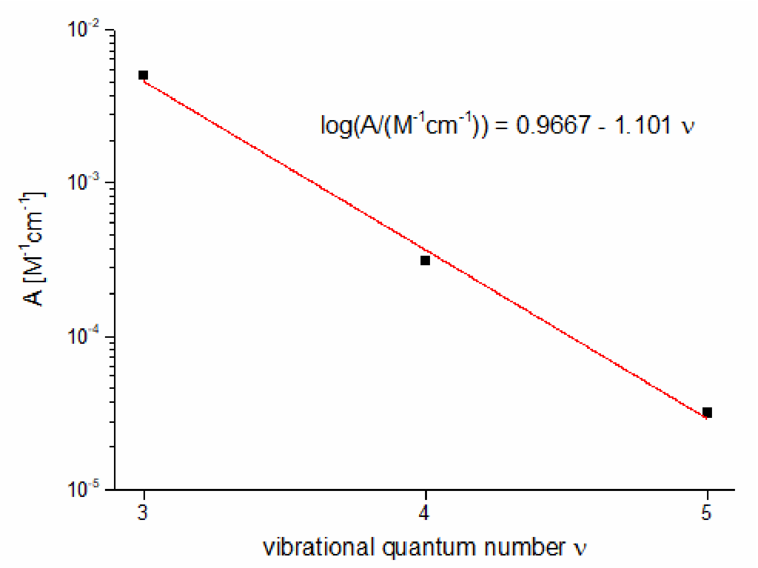

Figure S31. Semi-logarithmic plot of the amplitudes A of the Gaussians describing the C-D oscillators vs. the vibrational quantum number ν in CD_3CN and linear fit (solid red).

The other two Gaussian parameters (x_c and σ) necessary for the construction of the higher C-D overtone bands were estimated using the published data. The x_c values were calculated using the known Morse parameters for liquid CD_3CN (fundamental $\tilde{\nu}_0$ = 2175 cm^{-1} and anharmonicity x = 30.5 cm^{-1}),[S30] whereas the Gaussian widths for the required overtones σ were estimated to be equal to the corresponding C-D overtone band widths for aromatic C-D oscillators.[S29,S31] The properties of the

measured and extrapolated Gaussians are summarized in Table S4 and were used for the SOI calculations (vide infra).

Table S4. Parameters of the Gaussians (x_c: wavenumber of the maximum; σ: Gaussian width; *A*: amplitude) describing the C-D-oscillators in CD_3CN.

ν	maximum x_c / cm^{-1}	σ / cm^{-1}	A / $M^{-1}cm^{-1}$
3[a]	-	-	$5.01 \cdot 10^{-3}$
4[a]	-	-	$3.11 \cdot 10^{-4}$
5	-	-	$3.15 \cdot 10^{-5}$
6	12369[b]	129.05[c]	$2.29 \cdot 10^{-6}$ [d]
7	14217[b]	154.35[c]	$1.82 \cdot 10^{-7}$ [d]

[a] Based on Gaussian fits of measured data.
[b] Calculated using $x_c = \nu \cdot 2175\ cm^{-1} - \nu(\nu+1)\cdot 30.5\ cm^{-1}$ from ref. S30.
[c] Calculated using $\sigma = \nu \cdot 25\ cm^{-1} - 22.8\ cm^{-1}$ from ref. S29.
[d] Extrapolated using the equation $\log(A/(M^{-1}cm^{-1})) = 0.9667 - \nu \cdot 1.101$, obtained by linear fitting of log(A) vs. vibrational quantum number ν (obtained by Gaussian fitting for the overtones ν = 3 to 5, see Figures S28–S30).

The integrand functions for the SOIs between the chromium complex emission and C-(H/D) overtones in the isotopologic bulk acetonitriles were constructed according to the mathematical definition of the SOIs:

$$\mathrm{SOI} = \int I_{norm}(\tilde{\nu}) \cdot \varepsilon(\tilde{\nu}) \cdot \tilde{\nu}^{-4} d\tilde{\nu}$$

with I_{norm} being the chromium emission spectrum normalized to unit area and ε the decimal molar vibrational absorption coefficient (see extracted and extrapolated absorption spectra above), both expressed in the wavenumber scale $\tilde{\nu}$. The integrand functions were generated with a set of data points with a step size of 1 cm^{-1} (Figures S32 and S33). Numerical integration of the integrand functions (OriginPro 9.0) gave the corresponding values for the SOIs.

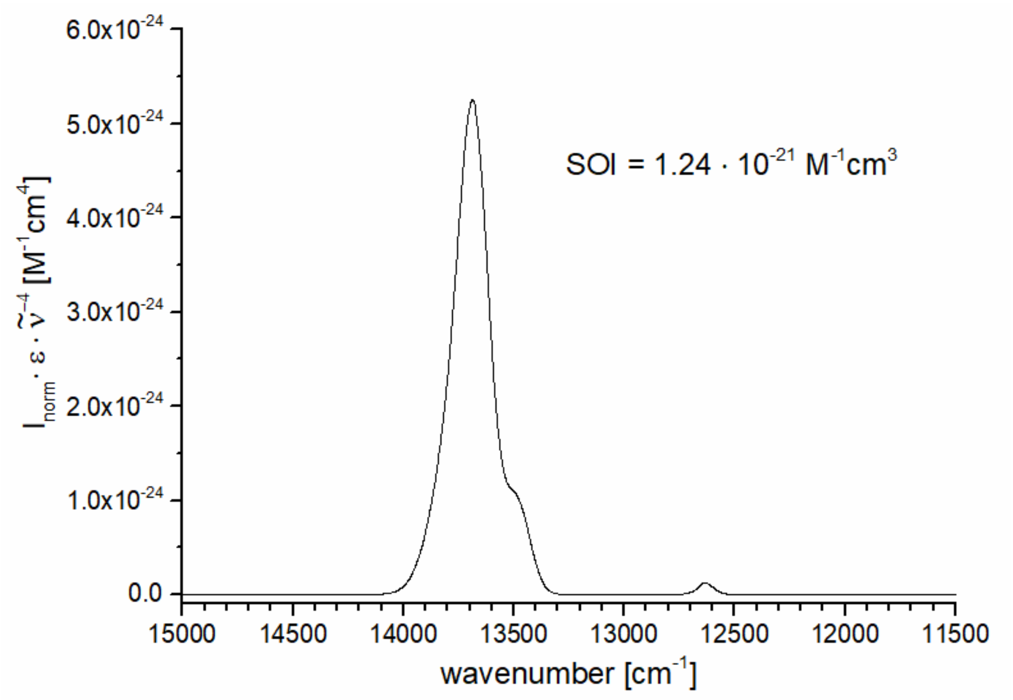

Figure S32. Integrand function of the spectral overlap integral (SOI) for chromium emission band in $[Cr(tpe)_2]^{3+}$ and the measured fourth overtone C-H ($\nu = 5$) and the measured third combination region of bulk CH_3CN.

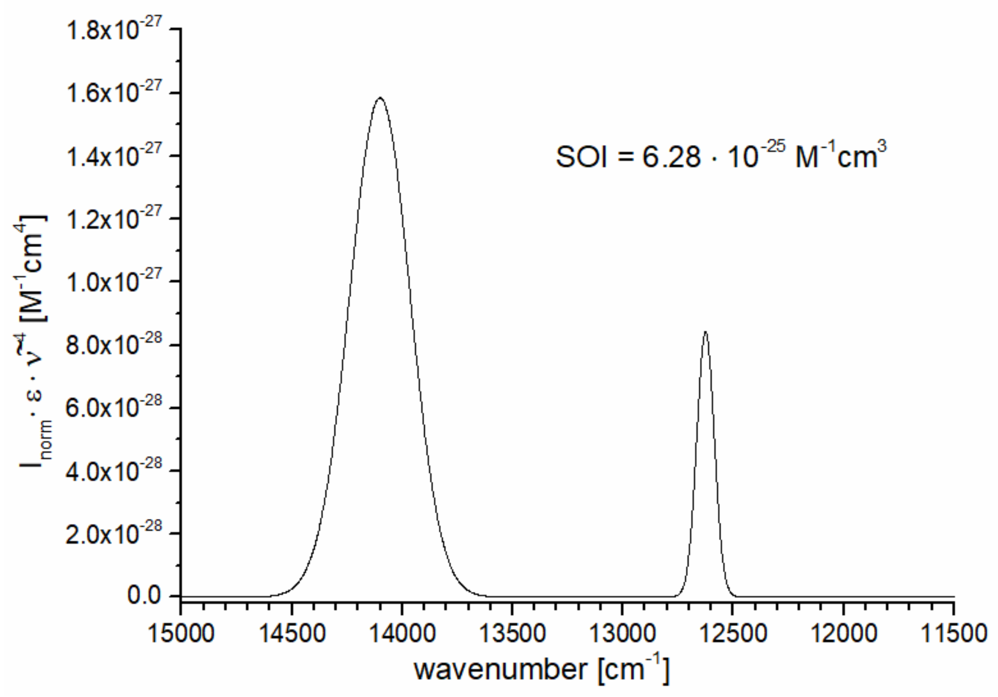

Figure S33. Integrand function of the spectral overlap integral (SOI) for chromium emission band in $[Cr(tpe)_2]^{3+}$ and the extrapolated fifth and sixth overtone C-D ($\nu = 6$ and $\nu = 7$) of bulk CD_3CN.

Based on the obtained SOIs, the ratio of the non-radiative deactivation rates of chromium excited states in $[Cr(tpe)_2]^{3+}$ in the acetonitrile isotopologues is estimated as:[S31]

$$\frac{k_{nr}(CH_3CN)}{k_{nr}(CD_3CN)} = \frac{SOI(CH_3CN)}{SOI(CD_3CN)} = \frac{1.24 \cdot 10^{-21}\ M^{-1}cm^3}{6.28 \cdot 10^{-25}\ M^{-1}cm^3} = 1.97 \cdot 10^3$$

This ratio would suggest a massive reduction in multiphonon relaxation rates and a concomitant increase in luminescence intensity and lifetimes by exchanging CH_3CN versus CD_3CN. Yet, this is not observed. For a discussion, see main text.

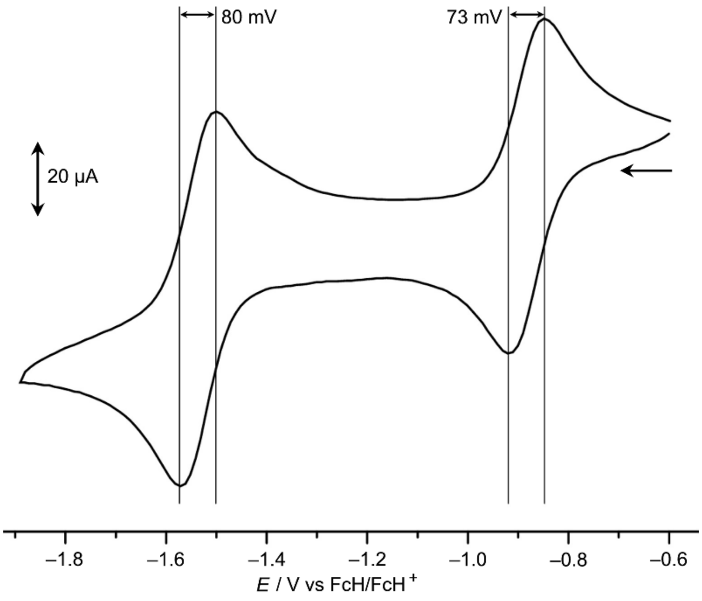

Figure S34. Cyclic voltammogram of $[Cr(tpe)_2][BF_4]_3$ (CH_3CN, $[N^nBu_4][BF_4]$, $T = 293$ K).

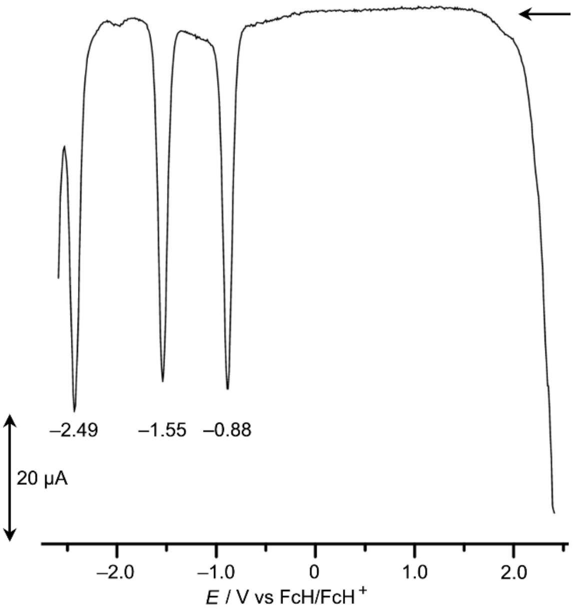

Figure S35. Square wave voltammogram of $[Cr(tpe)_2][BF_4]_3$ (CH_3CN, $[N^nBu_4][BF_4]$, $T = 293$ K).

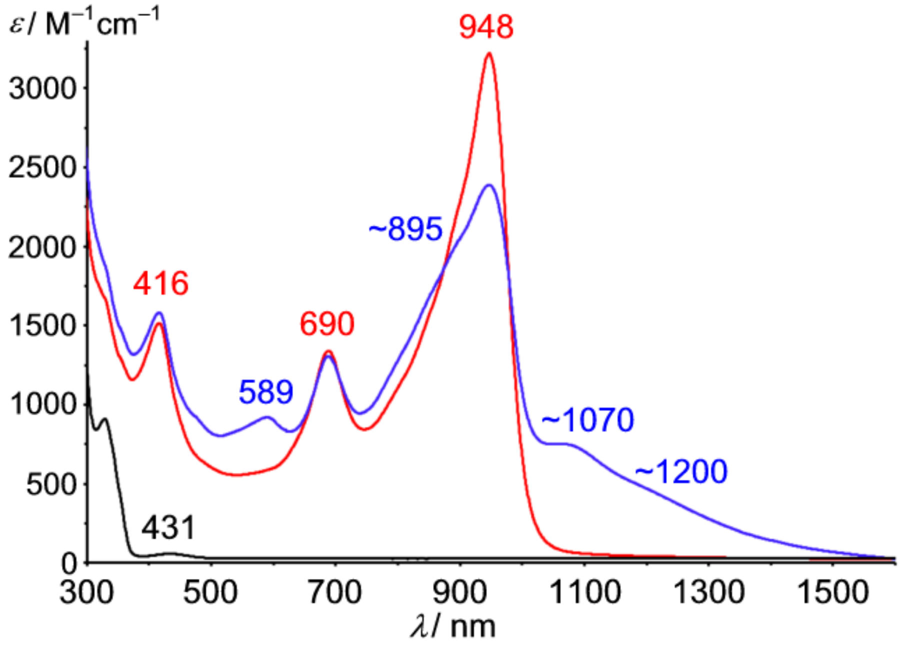

Figure S36. UV/Vis/NIR absorption spectra (CH_3CN, $[N^nBu_4][BF_4]$, T = 293 K) of $[Cr(tpe)_2][BF_4]_3$ (black), after the first reduction (red) and approximately after the second reduction (blue) in an OTTLE cell.

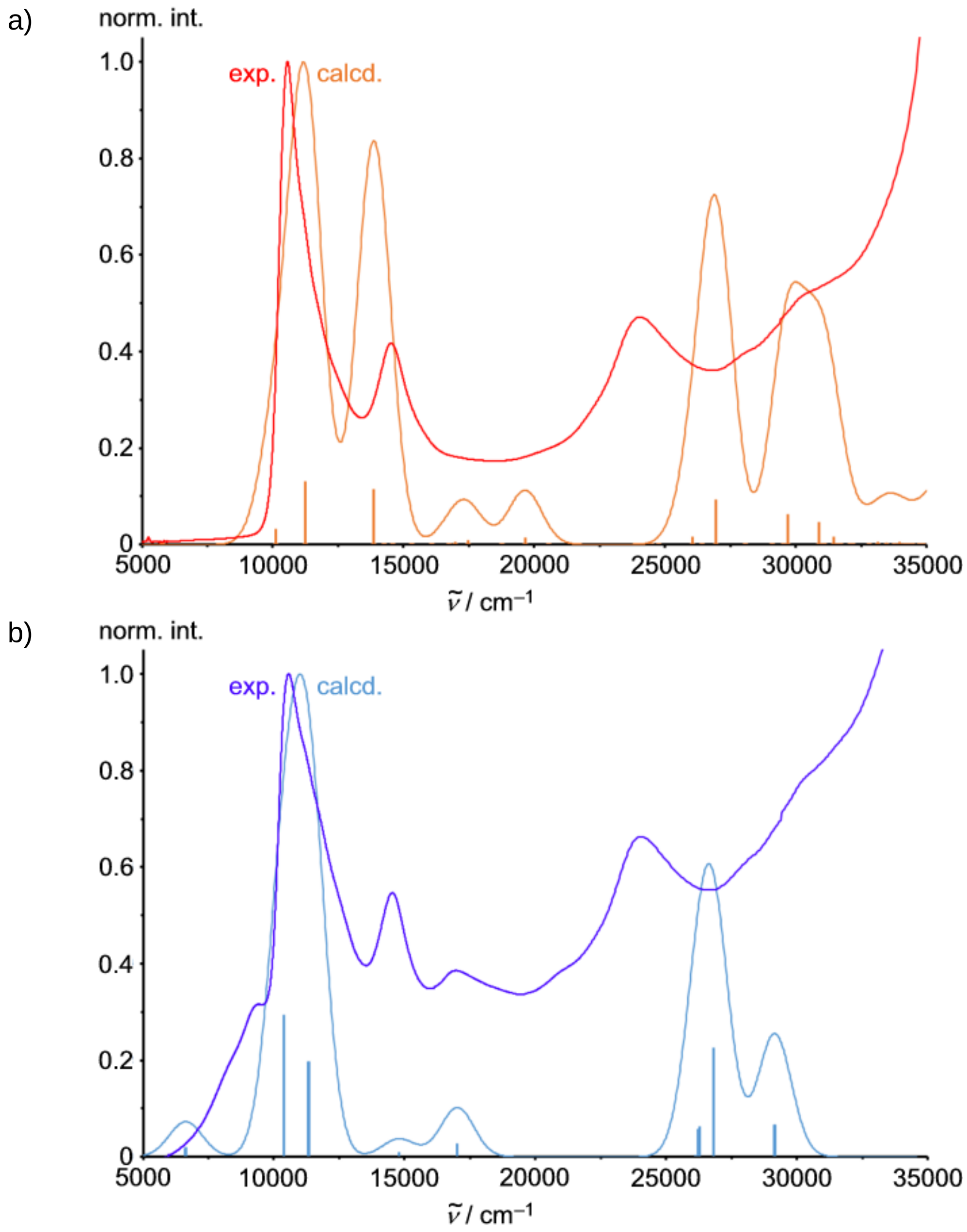

Figure S37. a) UV/Vis/NIR spectrum of $[Cr(tpe)_2][BF_4]_3$ after the first reduction in an OTTLE cell (CH_3CN, $[N^nBu_4][BF_4]$, T = 293 K) and TD-DFT calculated 50 vertical transitions of geometry optimized $[Cr(tpe)_2]^{2+}$ (S = 1). Stick spectrum was approximated by Gaussian bands with FWHM of 1500 cm^{-1}. b) UV/Vis/NIR spectrum of $[Cr(tpe)_2][BF_4]_3$ approximately after the second reduction in an OTTLE cell (CH_3CN, $[N^nBu_4][BF_4]$, T = 293 K) and TD-DFT calculated 50 vertical transitions of geometry optimized $[Cr(tpe)_2]^{+}$ (S = ½). The stick spectrum was approximated by Gaussian bands with FWHM of 1500 cm^{-1}).

Figure S38. a) Emission spectra of $[Cr(tpe)_2][BF_4]_3$ in CH_3CN during titration with azulene and b) corresponding Stern-Volmer analysis; $K_{SV} = 41.7\times10^3$ M^{-1}; $R^2 = 0.9887$.

a)

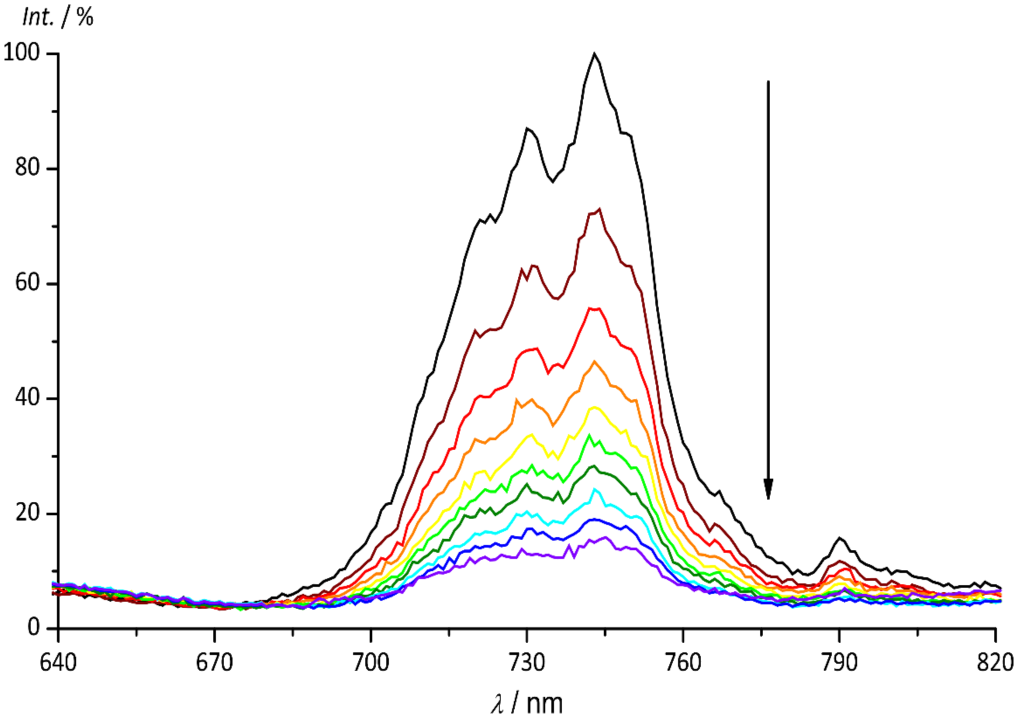

b)

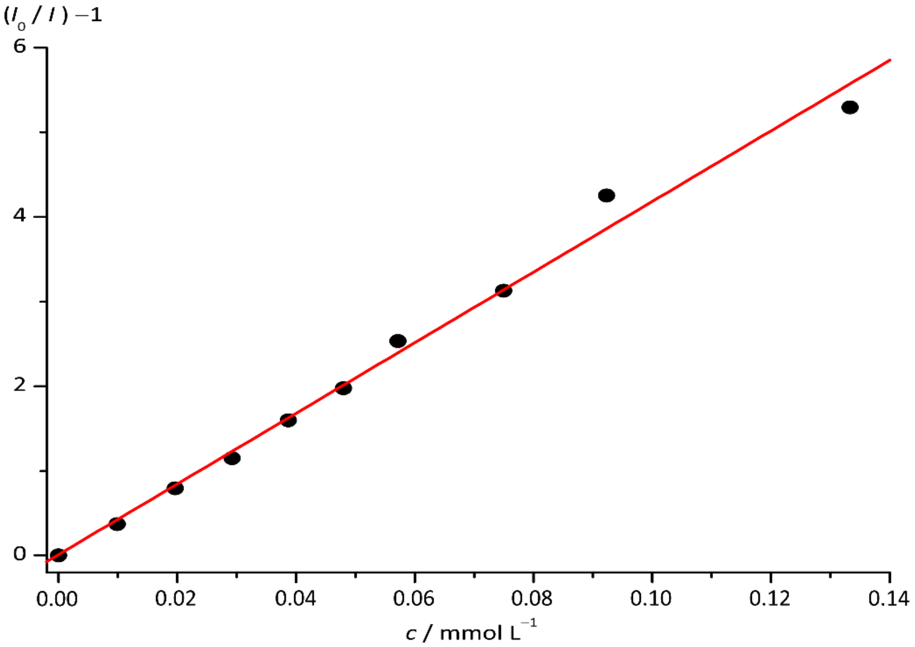

Figure S39. a) Emission spectra of $[Cr(tpe)_2][BF_4]_3$ in CH_3CN during titration with tri(*n*-butyl)amine and b) corresponding UV/Vis/NIR spectra showing the formation of $[Cr(tpe)_2]^{2+}$.

a)

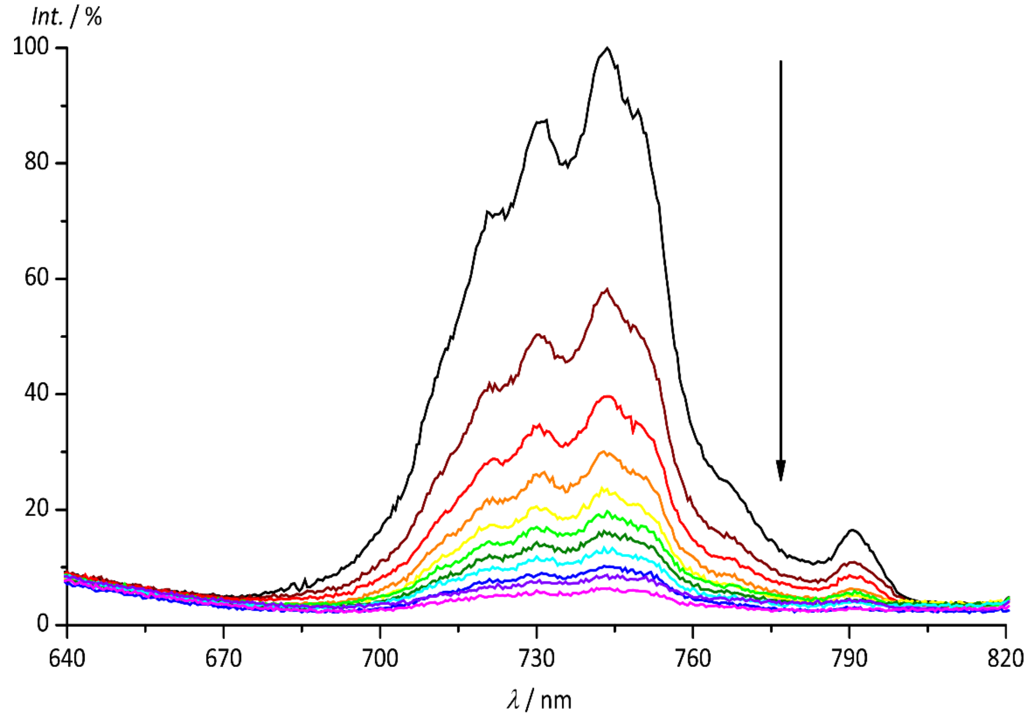

b)

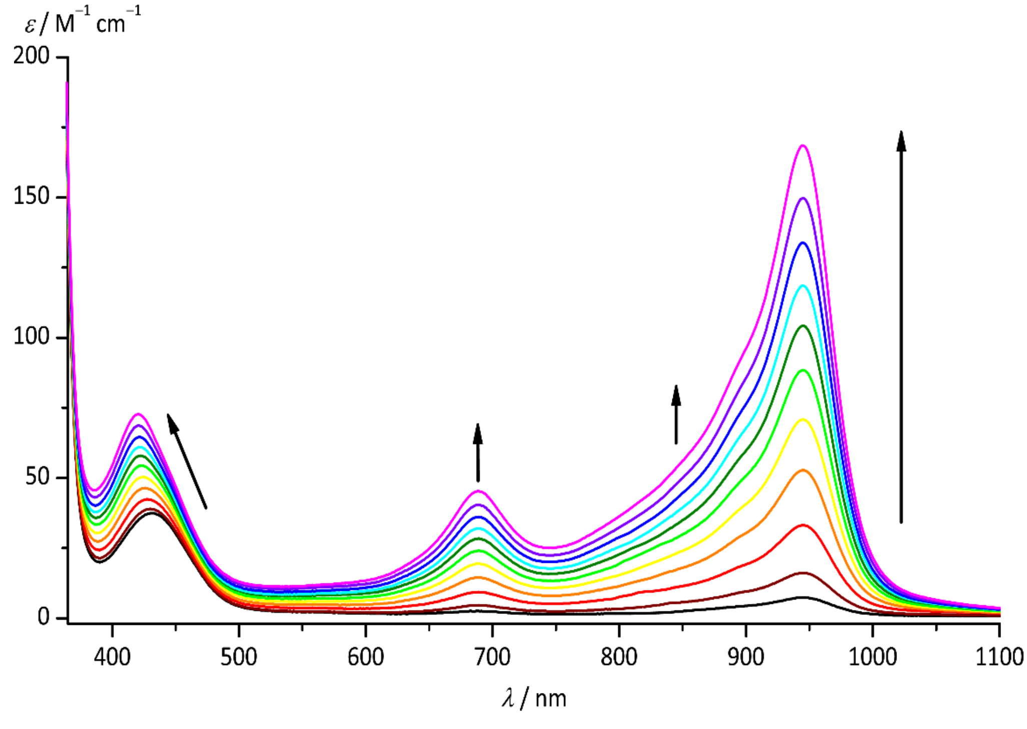

Cartesian coordinates of geometry optimized $[Cr(tpe)_2]^{3+}$ (quartet)

24	1.762771000	1.606228000	7.381007000
7	2.830770000	-0.144294000	7.240533000
7	3.574703000	2.576281000	7.360545000
7	1.999852000	1.450063000	9.414141000
6	2.282182000	1.228497000	12.141658000
1	2.407825000	1.137064000	13.211060000
6	4.347961000	0.830627000	8.934733000
6	4.925456000	4.374585000	6.567885000
1	5.009734000	5.258080000	5.953904000
6	5.818187000	2.759259000	8.095045000
1	6.641381000	2.392373000	8.681911000
6	5.985648000	3.902130000	7.325487000
1	6.937537000	4.413672000	7.322873000
6	3.734575000	3.682390000	6.612350000
1	2.880694000	4.010291000	6.041053000
6	3.195355000	1.095685000	9.924119000
6	1.059404000	1.593406000	11.600656000
1	0.198357000	1.797327000	12.218341000
6	0.958789000	1.693926000	10.229930000
1	0.029109000	1.973827000	9.760494000
6	3.355162000	0.979524000	11.297150000
1	4.303831000	0.698371000	11.718436000
6	5.617490000	0.450082000	9.700725000
1	5.454435000	-0.447613000	10.290063000
1	5.912650000	1.250249000	10.373200000
1	6.438000000	0.262482000	9.014423000
6	3.940119000	-0.313864000	7.985653000
6	4.672431000	-1.487289000	7.880572000
1	5.561553000	-1.634504000	8.467456000
6	4.258268000	-2.484912000	7.009139000
1	4.829710000	-3.398427000	6.927396000
6	3.113048000	-2.295775000	6.251873000
1	2.753137000	-3.043865000	5.562343000
6	2.426108000	-1.109847000	6.396155000
1	1.530843000	-0.913869000	5.827767000
6	4.595929000	2.103517000	8.101120000
7	0.696087000	3.357379000	7.521995000
7	-0.049255000	0.636280000	7.401840000
7	1.523873000	1.761864000	5.347984000
6	1.237859000	1.979350000	2.620485000
1	1.110769000	2.069156000	1.551117000
6	-0.823540000	2.382905000	5.829598000
6	-1.400067000	-1.161356000	8.195807000
1	-1.483852000	-2.045396000	8.809077000
6	-2.294516000	0.456196000	6.672047000
1	-3.118584000	0.824433000	6.087263000
6	-2.461529000	-0.687034000	7.441181000
1	-3.413989000	-1.197502000	7.445598000
6	-0.208554000	-0.470337000	8.149390000
1	0.646375000	-0.799644000	8.718321000
6	0.327725000	2.115777000	4.839094000
6	2.461322000	1.615108000	3.160374000
1	3.321517000	1.410118000	2.541856000
6	2.563695000	1.516592000	4.531110000
1	3.493936000	1.237063000	4.999662000

6	0.166097000	2.229852000	3.466096000
1	-0.783092000	2.510543000	3.045661000
6	-2.093597000	2.764279000	5.064868000
1	-1.930302000	3.661405000	4.474741000
1	-2.390443000	1.963945000	4.393344000
1	-2.913124000	2.953188000	5.752049000
6	-0.414013000	3.527356000	6.778089000
6	-1.145703000	4.701108000	6.883898000
1	-2.035236000	4.848806000	6.297773000
6	-0.730106000	5.698635000	7.754757000
1	-1.301065000	6.612402000	7.837066000
6	0.415789000	5.508997000	8.510876000
1	0.776788000	6.257006000	9.199928000
6	1.102166000	4.322831000	8.365854000
1	1.998506000	4.127165000	8.932734000
6	-1.071558000	1.110548000	6.663726000

Cartesian coordinates of geometry optimized $[Cr(tpe)_2]^{2+}$ (triplet)

24	1.762401000	1.606268000	7.381618000
7	2.822961000	-0.118886000	7.246885000
7	3.580216000	2.587258000	7.359208000
7	2.000140000	1.453165000	9.414812000
6	2.278355000	1.185517000	12.150257000
1	2.402419000	1.079833000	13.218706000
6	4.350078000	0.844738000	8.937164000
6	4.944986000	4.370987000	6.547859000
1	5.032580000	5.251609000	5.929500000
6	5.833203000	2.758446000	8.078059000
1	6.657104000	2.389794000	8.663185000
6	6.008026000	3.894138000	7.299974000
1	6.964002000	4.398479000	7.289250000
6	3.747213000	3.689660000	6.609901000
1	2.886739000	4.021380000	6.050191000
6	3.195477000	1.097469000	9.928105000
6	1.056784000	1.558276000	11.608680000
1	0.194010000	1.753150000	12.227783000
6	0.962480000	1.687256000	10.239109000
1	0.037291000	1.982751000	9.769728000
6	3.352260000	0.955997000	11.301502000
1	4.301872000	0.672893000	11.720185000
6	5.621502000	0.468616000	9.703175000
1	5.463034000	-0.428380000	10.294866000
1	5.916572000	1.270402000	10.374611000
1	6.442682000	0.279876000	9.017491000
6	3.938609000	-0.301741000	7.992349000
6	4.666410000	-1.480389000	7.896904000
1	5.553710000	-1.623685000	8.488537000
6	4.257092000	-2.487228000	7.033121000
1	4.825534000	-3.403062000	6.957912000
6	3.111868000	-2.290116000	6.272769000
1	2.749403000	-3.039698000	5.584922000
6	2.429088000	-1.101179000	6.405807000
1	1.537284000	-0.908070000	5.830717000
6	4.602107000	2.113577000	8.096955000
7	0.702123000	3.331533000	7.516586000
7	-0.055403000	0.625265000	7.404141000
7	1.524240000	1.759277000	5.348418000
6	1.245436000	2.026866000	2.613021000
1	1.121155000	2.132566000	1.544599000
6	-0.825482000	2.368029000	5.826602000
6	-1.420024000	-1.158680000	8.215271000
1	-1.507512000	-2.039445000	8.833442000
6	-2.308497000	0.454192000	6.685583000
1	-3.132469000	0.823004000	6.100656000
6	-2.483190000	-0.681674000	7.463435000
1	-3.439157000	-1.186032000	7.474197000
6	-0.222283000	-0.477299000	8.153229000
1	0.638258000	-0.809091000	8.712779000
6	0.328869000	2.115194000	4.835394000
6	2.467028000	1.653791000	3.154322000
1	3.329615000	1.458660000	2.535043000
6	2.561571000	1.524840000	4.523877000
1	3.486823000	1.229178000	4.993021000

6	0.171788000	2.256661000	3.462022000
1	-0.777878000	2.539972000	3.043603000
6	-2.097035000	2.744322000	5.060881000
1	-1.938613000	3.641410000	4.469317000
1	-2.392211000	1.942666000	4.389336000
1	-2.918098000	2.932924000	5.746738000
6	-0.413788000	3.514383000	6.771513000
6	-1.141706000	4.692926000	6.867403000
1	-2.029200000	4.836211000	6.276062000
6	-0.732228000	5.699701000	7.731182000
1	-1.300767000	6.615446000	7.806728000
6	0.413327000	5.502650000	8.491048000
1	0.776004000	6.252203000	9.178817000
6	1.096196000	4.313821000	8.357564000
1	1.988402000	4.121056000	8.932183000
6	-1.077403000	1.099070000	6.666644000

Cartesian coordinates of geometry optimized $[Cr(tpe)_2]^+$ (doublet)

24	1.762423000	1.606357000	7.381618000
7	2.807608000	-0.121602000	7.231621000
7	3.542356000	2.571934000	7.354993000
7	1.983413000	1.453542000	9.386812000
6	2.274256000	1.236047000	12.145636000
1	2.400994000	1.148099000	13.215267000
6	4.334773000	0.834002000	8.926777000
6	4.911342000	4.376345000	6.561578000
1	4.992564000	5.260842000	5.946344000
6	5.805289000	2.762513000	8.082882000
1	6.628232000	2.391243000	8.669101000
6	5.983295000	3.908177000	7.315142000
1	6.935914000	4.418429000	7.309628000
6	3.720293000	3.687325000	6.608163000
1	2.864114000	4.015384000	6.040177000
6	3.183570000	1.099987000	9.915421000
6	1.049692000	1.597838000	11.592868000
1	0.186384000	1.802645000	12.209372000
6	0.946469000	1.695105000	10.223487000
1	0.016537000	1.972263000	9.752677000
6	3.342122000	0.988165000	11.290169000
1	4.293917000	0.708879000	11.708073000
6	5.605044000	0.452851000	9.691889000
1	5.443900000	-0.444084000	10.283724000
1	5.904098000	1.252525000	10.363968000
1	6.426274000	0.262817000	9.006216000
6	3.925852000	-0.309250000	7.978949000
6	4.654062000	-1.486695000	7.873917000
1	5.541272000	-1.633168000	8.465577000
6	4.247986000	-2.491496000	7.002876000
1	4.817127000	-3.406657000	6.922733000
6	3.099812000	-2.288981000	6.243699000
1	2.737538000	-3.035330000	5.551491000
6	2.414736000	-1.103338000	6.385728000
1	1.520683000	-0.904326000	5.816293000
6	4.582276000	2.105685000	8.093766000
7	0.717281000	3.334326000	7.531654000
7	-0.017446000	0.640580000	7.408405000
7	1.541141000	1.759176000	5.376496000
6	1.249706000	1.976393000	2.617682000
1	1.122738000	2.064237000	1.548071000
6	-0.810240000	2.378531000	5.836908000
6	-1.386133000	-1.163954000	8.201994000
1	-1.467192000	-2.048514000	8.817165000
6	-2.280556000	0.449979000	6.681079000
1	-3.103661000	0.821274000	6.095103000
6	-2.458312000	-0.695742000	7.448780000
1	-3.410918000	-1.206018000	7.454525000
6	-0.195087000	-0.474912000	8.155199000
1	0.661307000	-0.802982000	8.722877000
6	0.340819000	2.112563000	4.848082000
6	2.474429000	1.614790000	3.170240000
1	3.337642000	1.410024000	2.553588000
6	2.577919000	1.517653000	4.539597000
1	3.508006000	1.240710000	5.010228000

6	0.181997000	2.224246000	3.473357000
1	-0.769910000	2.503401000	3.055616000
6	-2.080640000	2.759638000	5.071987000
1	-1.919631000	3.656603000	4.480158000
1	-2.379746000	1.959961000	4.399935000
1	-2.901787000	2.949597000	5.757778000
6	-0.401248000	3.521791000	6.784708000
6	-1.129700000	4.699057000	6.890096000
1	-2.017158000	4.845366000	6.298769000
6	-0.723559000	5.703873000	7.761092000
1	-1.292914000	6.618874000	7.841548000
6	0.424984000	5.501594000	8.519776000
1	0.787388000	6.248025000	9.211831000
6	1.110313000	4.316139000	8.377402000
1	2.004885000	4.117514000	8.946171000
6	-1.057554000	1.106832000	6.669929000

References

[S1] A. Santoro, C. Sambiagio, P. C. McGowan, M. A. Halcrow, *Dalton Trans.* **2015**, *44*, 1060–1069.

[S2] G. R. Fulmer, A. J. M. Miller, N. H. Sherden, H. E. Gottlieb, A. Nudelman, B. M. Stoltz, J. E. Bercaw, K. I. Goldberg, *Organometallics* **2010**, *29*, 2176–2179.

[S3] C. Würth, J. Pauli, C. Lochmann, M. Spieles, U. Resch-Genger, *Anal. Chem.* **2012**, *84*, 1345–1352.

[S4] C. Würth, M.-G. González, R. Niessner, U. Panne, C. Haisch, U. Resch-Genger, *Talanta* **2012**, *90*, 30–37.

[S5] C. Würth, D. Geissler, T. Behnke, M. Kaiser, U. Resch-Genger, *Anal. Bioanal. Chem.* **2015**, *407*, 59–78.

[S6] M. Zimmer, F. Rupp, P. Singer, F. Walz, F. Breher, W. Klopper, R. Diller, M. Gerhards, *Phys. Chem. Chem. Phys.* **2015**, *17*, 14138–14144.

[S7] F. Bäppler, M. Zimmer, F. Dietrich, M. Gruppe, M. Wallesch, D. Volz, S. Bräse, M. Gerhards, R. Diller, *Phys. Chem. Chem. .Phys.* **2017**, *19*, 29438–29448.

[S8] M. Zimmer, F. Dietrich, D. Volz, S. Bräse, M. Gerhards, *ChemPhysChem* **2017**, *18*, 3023–3029.

[S9] STOE & Cie, X-Red, STOE & Cie, Darmstadt, Germany **2002**.

[S10] R. H. Blessing, *Acta Crystallogr. Sect. A* **1995**, *51*, 33–38.

[S11] A. L. Spek, *Acta Crystallogr. Sect. D* **2009**, *65*, 148–55.

[S12] G. M. Sheldrick, SHELXL-2014/7, University of Göttingen, Göttingen, Germany, **2014**.

[S13] G. M. Sheldrick, *Acta Crystallogr. Sect. A* **2015**, *71*, 3–8.

[S14] F. Neese, *WIREs Comput. Mol. Sci.* **2012**, *2*, 73–78.

[S15] F. Neese, F. Wennmohs, A. Hansen, U. Becker, *Chem. Phys.* **2009**, *356*, 98–109.

[S16] R. Izsák, F. Neese, *J. Chem. Phys.* **2011**, *135*, 144105.

[S17] A. D. Becke, *J. Chem. Phys.* **1993**, *98*, 5648–5652.

[S18] F. Weigend, R. Ahlrichs, *Phys. Chem. Chem. Phys.* **2005**, *7*, 3297–3305.

[S19] F. Weigend, *Phys. Chem. Chem. Phys.* **2006**, *8*, 1057–1065.

[S20] D. A. Pantazis, X.-Y. Chen, C. R. Landis, F. Neese, *J. Chem. Theory Comput.* **2008**, *4*, 908–919.

[S21] E. van Lenthe, E. J. Baerends, J. G. Snijders, *J. Chem. Phys.* **1993**, *99*, 4597–4610.

[S22] S. Grimme, J. Antony, S. Ehrlich, H. Krieg, *J. Chem. Phys.* **2010**, *132*, 154104.

[S23] S. Grimme, S. Ehrlich, L. Goerigk, *J. Comput. Chem.* **2011**, *32*, 1456–1465.

[S24] V. Barone, M. Cossi, *J. Phys. Chem. A* **1998**, *102*, 1995–2001.

[S25] S. Otto, M. Grabolle, C. Förster, C. Kreitner, U. Resch-Genger, K. Heinze, *Angew. Chem.* **2015**, *127*, 11735-11739; *Angew. Chem. Int. Ed.* **2015**, *54*, 11572–11576;

[S26] S. Lenz, H. Bamberger, P. P. Hallmen, Y. Thiebes, S. Otto, K. Heinze, J. van Slageren, *Phys. Chem. Chem. Phys.* **2019**, *21*, 6976–6983.

[S27] a) U. Casellato, R. Graziani, R. P. Bonomo, A. J. Di Bilio, *J. Chem. Soc. Dalton Trans.* **1991**, 23–31; b) R. P. Bonomo, A. J. DiBilio, F. *Chem. Phys.* **1991**, *151*, 323–333.

[S28] M. K. Ahmed, B. R. Henry, *J. Chem. Phys.* **1987**, *87*, 3724.

[S29] C. Wang, S. Otto, M. Dorn, E. Kreidt, J. Lebon, L. Srsan, P. Di Martino-Fumo, M. Gerhards, U. Resch-Genger, M. Seitz, K. Heinze, *Angew. Chem.* **2018**, *130*, 1125–1130; *Angew. Chem. Int. Ed.* **2018**, *57*, 1112–1116.

[S30] M. K. Ahmed, B. R. Henry, *Can. J. Chem.* **1988**, *66*, 628–632.

[S31] C. Doffek, N. Alzakhem, C. Bischof, J. Wahsner, T. Güden-Silber, J. Lügger, C. Platas-Iglesias, M. Seitz, *J. Am. Chem. Soc.* **2012**, *134*, 16413–16423.
